# Bayesian variable selection with a pleiotropic loss function in Mendelian randomization

**DOI:** 10.1002/sim.9109

**Published:** 2021-06-21

**Authors:** Apostolos Gkatzionis, Stephen Burgess, David V. Conti, Paul J. Newcombe

**Affiliations:** 1 MRC Biostatistics Unit, University of Cambridge, Cambridge, UK; 2 MRC Integrative Epidemiology Unit, University of Bristol, Bristol, UK; 3 Department of Public Health and Primary Care, School of Clinical Medicine, University of Cambridge, Cambridge, UK; 4 Department of Preventive Medicine, Keck School of Medicine, University of Southern California, Los Angeles, California

**Keywords:** general Bayesian inference, instrumental variables, Mendelian randomization, pleiotropy, variable selection

## Abstract

Mendelian randomization is the use of genetic variants as instruments to assess the existence of a causal relationship between a risk factor and an outcome. A Mendelian randomization analysis requires a set of genetic variants that are strongly associated with the risk factor and only associated with the outcome through their effect on the risk factor. We describe a novel variable selection algorithm for Mendelian randomization that can identify sets of genetic variants which are suitable in both these respects. Our algorithm is applicable in the context of two-sample summary-data Mendelian randomization and employs a recently proposed theoretical extension of the traditional Bayesian statistics framework, including a loss function to penalize genetic variants that exhibit pleiotropic effects. The algorithm offers robust inference through the use of model averaging, as we illustrate by running it on a range of simulation scenarios and comparing it against established pleiotropy-robust Mendelian randomization methods. In a real-data application, we study the effect of systolic and diastolic blood pressure on the risk of suffering from coronary heart disease (CHD). Based on a recent large-scale GWAS for blood pressure, we use 395 genetic variants for systolic and 391 variants for diastolic blood pressure. Both traits are shown to have significant risk-increasing effects on CHD risk.

## 1 | INTRODUCTION

Mendelian randomization provides a framework for probing questions of causality from observational data using genetic variants. It applies the theory of instrumental variable analysis from the causal inference literature, using genetic variants associated with the risk factor as instruments. Mendelian randomization relies on the idea that, since genetic variants are randomly inherited and fixed at conception, they should be uncorrelated with potential confounders of the relationship between the risk factor and outcome and are therefore suitable to use as instruments. This approach has received much attention since the seminal paper of Davey Smith and Ebrahim,^1^ and has led to a number of influential results over the last decade addressing a variety of aetiological questions.^2^ For example, in coronary heart disease (CHD), Mendelian randomization has been used to strengthen the evidence for a causal role of lipoprotein(a),^3^ but to weaken the case for C-reactive protein.^4^

Formally, Mendelian randomization relies on three basic assumptions:

1. There is no confounding in the association between the genetic variants and the outcome.
2. The genetic variants are associated with the risk factor of interest.
3. The genetic variants only influence the outcome via their association with the risk factor and not through alternative causal mechanisms.

**FIGURE 1:**
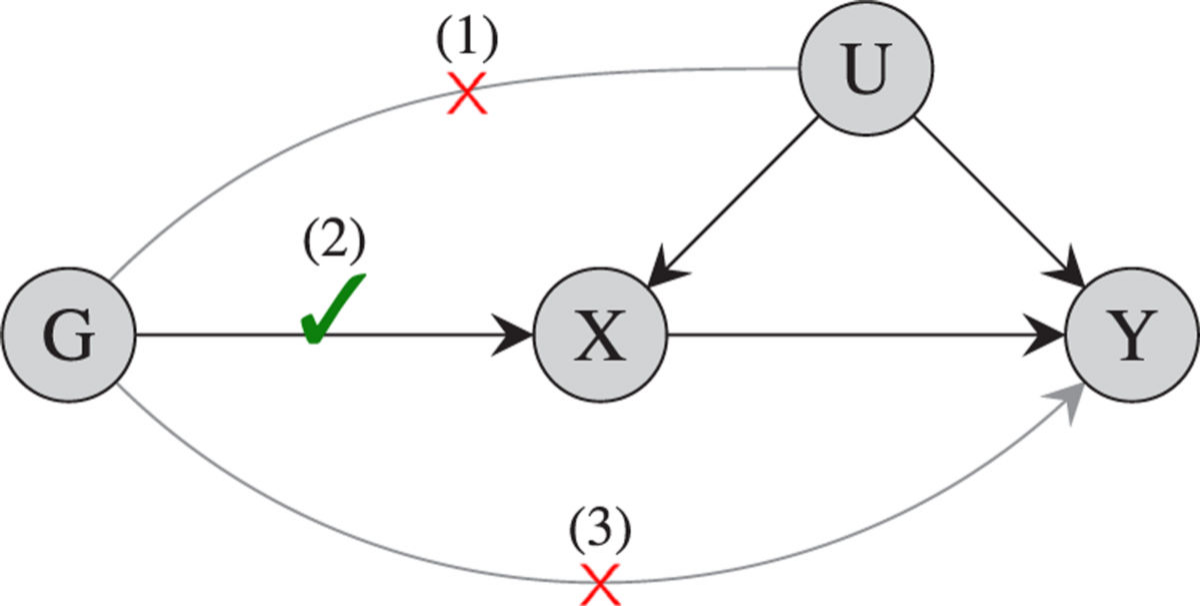
A causal diagram representation of the three assumptions of Mendelian randomization. Here, *X* represents the risk factor, *Y* the outcome, *G* the genetic instrument, and *U* denotes confounders of the *X* − *Y* relationship [Colour figure can be viewed at wileyonlinelibrary.com]

The three assumptions are shown in Figure 1. Under these assumptions, valid causal inferences can be made as to whether the risk factor affects the outcome.

As mentioned earlier, Assumption 1 is usually justified on the basis of Mendelian inheritance: an individual’s genotype is randomly assorted at conception and not influenced by external confounding factors. To ensure the validity of Assumption 2 Mendelian randomization analyses utilize the results of large consortia meta-GWAS, which use sample sizes of tens or hundreds of thousands of individuals to identify genetic variants robustly associated with a trait. In particular, many recent Mendelian randomization studies have taken a two-sample approach in which genetic associations with the target risk factor and with the outcome are assessed in separate datasets, in order to leverage their respective (and often mutually exclusive) meta-GWAS. However, these GWAS results are rarely available as individual-level data. Usually, only a set of summary statistics, such as univariate SNP-trait associations and corresponding standard errors (SEs), are reported. As a result, a large number of recent Mendelian randomization investigations rely on summarized data.

Assumption 3, often called the exclusion restriction or no-pleiotropy assumption, has received much attention in the recent literature. In practical applications, we typically do not know which genetic variants exhibit pleiotropic effects and there is need for methods to perform Mendelian randomization in the presence of pleiotropic variants. Traditional approaches include MR-Egger regression^5^ and median estimation,^6^ while several algorithms for pleiotropy-robust Mendelian randomization have been developed recently.^7–15^ Recent reviews and comparison of the various methods have also been conducted.^16,17^

In this article, we add to the relevant literature by proposing a new method for variable selection and causal effect estimation in Mendelian randomization. Our method is derived as an extension of the JAM algorithm (Joint Analysis of Marginal summary statistics).^18^ JAM was originally proposed for fine-mapping genetic regions. Similar to other recently proposed fine-mapping algorithms,^19,20^ JAM is designed to work with summary GWAS data. The algorithm performs variable selection to identify genetic variants robustly associated with the trait. Genetic correlations are taken into account by estimating them from a reference dataset such as 1000 Genomes or the UK Biobank. Variable selection is performed according to a Bayesian stochastic search algorithm, which can explore the complete space of causal configurations. Consequently, JAM is able to explore complex models with large numbers of variants, as recently demonstrated while fine-mapping dense genotype data for prostate cancer risk.^21^

We develop a novel model averaging variable selection algorithm for Mendelian randomization, which we call JAM-MR (JAM for Mendelian randomization). To do so, we modify JAM’s variable selection to downweight genetic variants which exhibit heterogeneous effects. We use the recently proposed framework of general Bayesian inference,^22^ in which the likelihood is augmented with a loss function before obtaining a Bayesian loss- posterior distribution. JAM’s likelihood is hence combined with a loss function that penalizes models containing variants with heterogeneous univariate causal effect estimates. Our algorithm performs variable selection and returns variant-specific posterior inclusion probabilities, which can be interpreted as probabilities of each variant being a valid instrument, and posterior model probabilities, which can subsequently be used to estimate the causal effect of interest by averaging across model-specific estimates. Uncertainty in which variants should be excluded on the basis of pleiotropy is reflected by averaging estimates over competing selections of instruments.

Conceptually, the JAM-MR algorithm has a number of attractive features. The use of Bayesian stochastic search has already proven useful in other areas of statistical genetics, such as fine-mapping^18,19^ and colocalization.^23^ The Bayesian paradigm allows us to incorporate prior information on the suitability of genetic variants as instruments into the analysis. It also allows us to model the uncertainty in genetic associations with the risk factor, which is often ignored by other approaches. JAM-MR also offers a natural framework for incorporating genetic correlations, when conducting Mendelian randomization with several genetic variants coming from the same gene region.

The performance of our algorithm is illustrated in a range of simulated datasets, as well as in a real-data application where we investigate the causal effect of systolic and diastolic blood pressure on the risk of CHD. We instrument blood pressure using a recently published large-scale meta-GWAS, which combined results across more than one million individuals, and therefore base our Mendelian randomization analysis on larger sample sizes and more genetic variants compared with previous studies in the literature. Our results strengthen the claim for a risk-increasing causal relationship between blood pressure traits and CHD risk.

## 2 | METHODS

### 2.1 | The JAM algorithm

#### 2.1.1 | Introduction

We start by providing a brief outline of the JAM algorithm. The reader is referred to the relevant paper^18^ for a more detailed description.

The JAM algorithm is primarily a tool for fine-mapping densely genotyped DNA regions. The standard fine-mapping question is as follows: given *P* correlated genetic variants *G*_1_, … *,G*_*P*_, identify which variants are causally and independently associated with a trait *X* of interest. The genetic variants are typically located in the same gene region. The main purpose of JAM is to perform variable selection for the genetic variants, given a set of GWAS summary statistics and an external dataset from which to estimate genetic correlations.

For individual *i*, let *g*_*i*_ = (*g*_*i*1_, … *,g*_*iP*_) and *x*_*i*_ be the allele counts and trait measurements, respectively, and denote *G* = (*g*_*ij*_) the genetic matrix. We assume that trait values and allele counts per variant have been centered. Typically, the “individual-level data” *x*_*i*_*,g*_*i*_ are not available in practice. Instead, we only have access to a set of univariate association estimates ${\hat{\beta }}_{Xj}$ between each variant and the trait, as well as the corresponding SEs ${\hat{s}}_{Xj}$.

#### 2.1.2 | The JAM model

JAM uses linear regression to model the trait: if all *P* genetic variants were assumed to be associated with *X*, the JAM algorithm would model the trait as

(1) $$x_{i}|\beta ,\sigma_{X}^{2},G=\sum_{j=1}^{P}g_{ij}\beta_{j}+\epsilon_{Xi},\;\epsilon_{Xi}∼N\left(0,\sigma_{X}^{2}\right),\;i=1,\ldots ,N_{1}.$$

In practice JAM implements variable selection, reflecting that, in many applications, only a subset of the variants should be used to model the trait. Let *𝛾* ∈ {0,1}^*P*^ be a vector of binary indicators, such that *𝛾*_*j*_ = 1 if variant *G*_*j*_ is included into the JAM model and *𝛾*_*j*_ = 0 if it is not. Moreover, let *R*_*𝛾*_ = {*j* ∈ {1, … *,P*} ∶ *𝛾*_*j*_ = 1} be the set of genetic variants included in the model, and *P*_*𝛾*_ =|*R*_*𝛾*_|be the number of variants in *R*_*𝛾*_. Equation (1) becomes

(2) $$x_{i}|\beta ,\sigma_{X}^{2},\gamma ,G=\sum_{j\in R_{\gamma }}g_{ij}\beta_{j}+\epsilon_{Xi},\;\epsilon_{Xi}∼N\left(0,\sigma_{X}^{2}\right).$$

Let *𝛽*_*𝛾*_ denote the subvector of *𝛽* containing only those entries *𝛽*_*j*_ for which *𝛾*_*j*_ = 1, *G*_*𝛾*_ be the *N*_1_ × *P*_*𝛾*_ submatrix of *G* containing only those columns *j* for which *𝛾*_*j*_ = 1 and *g*_*𝛾i*_ be the *i*th row of *G*_*𝛾*_. Equation (2) can be used to build a likelihood for the individual-level data $p\left(x|\beta_{\gamma },\sigma_{X}^{2},\gamma ,G_{\gamma }\right)=\prod_{i}p\left(x_{i}|\beta_{\gamma },\sigma_{X}^{2},\gamma ,g_{\gamma i}\right)$. One can also obtain the marginal model likelihood, $p\left(x|\gamma ,G_{\gamma }\right)=\int p\left(x|\beta_{\gamma },\sigma_{X}^{2},\gamma ,G_{\gamma }\right)d\beta_{\gamma }d\sigma_{X}^{2}$. JAM works by constructing summary-data approximations to these two likelihoods (see the following subsections).

#### 2.1.3 | Prior specification

The likelihood $p\left(x|\beta_{\gamma },\sigma_{X}^{2},\gamma ,G_{\gamma }\right)$ is complemented with a set of priors in order to perform Bayesian inference. For the genetic associations| *𝛽*_*𝛾*_, JAM uses a conjugate g-prior,

(3) $$\beta_{\gamma }|\sigma_{X}^{2},\gamma ∼N\left(0,\sigma_{X}^{2}\tau {\left(G_{\gamma }^{T}G_{\gamma }\right)}^{-1}\right),$$

where *𝜏* is a constant. By default, the algorithm sets *𝜏* = max(*P*^2^*,N*_1_), as has been previously recommended by various authors.^24,25^ The residual variance $\sigma_{X}^{2}$ is assigned its own conjugate prior, which is an Inverse-Gamma density,

(4) $$\sigma_{X}^{2}∼IG\left(a_{X},b_{X}\right),$$

for fixed *a*_*X*_, *b*_*X*_. Finally, JAM uses a Beta-Binomial prior on the space of all possible models,

(5) $$p(\gamma )=\frac{B\left(a_{\omega }+P_{\gamma },b_{\omega }+P-P_{\gamma }\right)}{B\left(a_{\omega },b_{\omega }\right)},$$

where *B*(*a,b*) denotes the Beta function. This is a binomial prior on the number of variants associated with *X*, with the prior proportion of causal variants treated as a hyperparameter and assigned its own Beta(*a*_*𝜔*_*,b*_*𝜔*_) prior. By default, *a*_*𝜔*_ = 1, *b*_*𝜔*_ = *P*, which corresponds to an expectation that a single variant will be associated with the trait. The Beta-Binomial prior assigns the same prior probability to models of equal size, and the same prior inclusion probability to different variants. In applications where prior information on the associations on specific variants with the risk factor is available, this information can be incorporated into JAM by partitioning the variants into subgroups and assigning a different prior inclusion probability to variants in each subgroup.

#### 2.1.4 | Posterior inference for the regression parameters

A Bayesian posterior distribution over all the parameters (model indicator, genetic associations and residual variance) can be obtained according to the standard principles of Bayesian inference:

(6) $$p\left(\beta_{\gamma },\sigma_{X}^{2},\gamma |x,G\right)\propto p\left(x|\beta_{\gamma },\sigma_{X}^{2},\gamma ,G\right)p\left(\beta_{\gamma }|\sigma_{X}^{2},\gamma \right)p\left(\sigma_{X}^{2}|\gamma \right)p(\gamma ).$$

Conditional on a particular combination of causal variants, posterior inference on the regression parameters $\beta_{\gamma },\sigma_{X}^{2}$ can be conducted using known results for Bayesian linear regression with conjugate priors, which yield

(7) $$\sigma_{X}^{2}|x,\gamma ∼IG\left(a_{X}+\frac{N_{1}}{2},b_{X}+\frac{s^{2}}{2}+\frac{{\hat{\beta }}_{\gamma }^{T}G_{\gamma }^{T}G_{\gamma }{\hat{\beta }}_{\gamma }}{2(\tau +1)}\right)$$

(8) $$\beta_{\gamma }|x,\sigma_{X}^{2},\gamma ∼N\left(\frac{\tau }{1+\tau }{\hat{\beta }}_{\gamma },\frac{\tau }{\tau +1}\sigma_{X}^{2}{\left(G_{\gamma }^{T}G_{\gamma }\right)}^{-1}\right)$$

where ${\hat{\beta }}_{\gamma }={\left(G_{\gamma }^{T}G_{\gamma }\right)}^{-1}G_{\gamma }^{T}x\text{ and }s^{2}={\left(x-G_{\gamma }{\hat{\beta }}_{\gamma }\right)}^{T}\left(x-G_{\gamma }{\hat{\beta }}_{\gamma }\right)$.

#### 2.1.5 | Posterior model selection

An advantage of the linear regression setting is that it allows for fast and efficient variable selection. The regression coefficients *𝛽*_*𝛾*_ and the residual variance $\sigma_{X}^{2}$ can be integrated out from the JAM likelihood $p\left(x|\beta_{\gamma },\sigma_{X}^{2},\gamma \right)$ to obtain the marginal model likelihood

(9) $$p(x|\gamma )\propto {\left(\tau +1\right)}^{-\frac{P_{\gamma }}{2}}{\left(2b_{\sigma }+S(\gamma )\right)}^{-a_{\sigma }-\frac{N_{1}}{2}},$$

where

(10) $$S(\gamma )=x^{T}x-\frac{\tau }{\tau +1}x^{T}G_{\gamma }{\left(G_{\gamma }^{T}G_{\gamma }\right)}^{-1}G_{\gamma }^{T}x.$$

This leads to the marginal model posterior, *p*(*𝛾*∣*x*) ∝ *p*(*x* ∣*𝛾*)*p*(*𝛾*). The normalizing constant of that density can be difficult to compute, as it would involve a summation over all possible models. To avoid this, JAM implements a reversible-jump MCMC algorithm,^26^ which only requires the posterior to be evaluated up to a normalizing constant. JAM implements a standard reversible-jump algorithm with addition, deletion and swapping of genetic variants as possible moves. The stochastic search algorithm allows exploration of an unrestricted model space, without the need to set limits on the maximum number of causal variants. Consequently, JAM is able to efficiently explore complex causal configurations among large numbers of genetic variants. Posterior model probabilities can be estimated by the proportion of iterations JAM spends in each model.

#### 2.1.6 | JAM with summarized data

One of JAM’s main advantages is that it does not require access to individual-level data. Variable selection is implemented according to Equations (9) and (10), which depend on the observed data *x*_*i*_*,g*_*i*_ only through the quantities *x*^*T*^*x*, *G*^*T*^*x* and *G*^*T*^*G*. These quantities are sufficient summary statistics for linear regression. In the setting of genome-wide association studies, $z=G^{T}x$ can be approximated^18^ by the univariate variant-trait association estimates ${\hat{\beta }}_{Xj},j=1,\ldots ,P$, as well as effect allele frequencies. In addition, note that $x^{T}x\approx \left(N_{1}-1\right){\hat{\sigma }}_{X}^{2}$ since we have assumed that trait values have been centered before implementing JAM. Here, ${\hat{\sigma }}_{X}^{2}$ is an estimator of the trait variance Var(*X*) measured in the GWAS for the trait *X*. This variance is sometimes reported by genetic association studies, but can be approximated using the SNP-trait associations, SEs and effect allele frequencies if it is not available.^27^ Finally, the matrix *G*^*T*^*G* models (*N*_1_ − 1 times) the genetic correlations between variants *G*_1_, … *,G*_*P*_ and can be approximated if a reference dataset, such as the 1000 Genomes dataset or the UK Biobank, is available. This yields the following summary-data approximation for *S*(*𝛾*):

(11) $$S(\gamma )\approx \left(N_{1}-1\right){\hat{\sigma }}_{X}^{2}-\frac{\tau }{\tau +1}{\hat{z}}^{T}{\left(G_{\gamma ,\text{ref}}^{T}G_{\gamma ,\text{ref}}\right)}^{-1}\hat{z}.$$

The model-specific marginal posteriors (7) and (8) can be approximated by summary GWAS data in a similar way.

#### 2.1.7 | Binary traits

The JAM algorithm assumes that the trait of interest is continuous, as is often the case for risk factors used in Mendelian randomization studies. JAM then relies on a linear modeling framework to relate univariate summary statistics to multivariate effects via the transformation $z=G^{T}x$ If the trait studied is binary, *G*^*T*^*x* can be derived by mapping univariate log-odds ratios to the univariate effects that would have been estimated if the binary trait was modeled by linear regression. The same strategy is employed in other linear-based summary data frameworks, including LDPred,^28^ to which we refer readers for a detailed description of this mapping.

### 2.2 | An extension of JAM for Mendelian randomization

#### 2.2.1 | Scope

We now describe our extension to the standard JAM algorithm that facilitates pleiotropy adjustment and accurate causal effect estimation in Mendelian randomization studies, which we call JAM-MR.

For the JAM-MR algorithm, we work in the context of two-sample summary-data Mendelian randomization. This framework allows researchers to leverage the power and large sample sizes of large consortia GWAS studies that are already available for many important traits, without the requirement that both the risk factor and the outcome should be measured on the same GWAS. It requires access to two different genetic association studies, one from which to obtain the SNP-risk factor summary statistics ${\hat{\beta }}_{Xj},{\hat{s}}_{Xj}$ and another study from which to obtain the SNP-outcome summary statistics ${\hat{\beta }}_{Yj},{\hat{S}}_{Yj}$. The coefficients ${\hat{\beta }}_{Xj},{\hat{\beta }}_{Yj}$ can be used to obtain variant-specific estimates of the causal effect *𝜃* according to a ratio formula,^29^

(12) $${\hat{\theta }}_{j}=\frac{{\hat{\beta }}_{Yj}}{{\hat{\beta }}_{Xj}},\;{\hat{s}}_{\theta j}=\text{ s}.\text{e}.\;\left({\hat{\theta }}_{j}\right)=\frac{{\hat{s}}_{Yj}}{{\hat{\beta }}_{Xj}}.$$

The genetic variants *G*_1_, … *,G*_*P*_ used in the JAM-MR algorithm can be either correlated or independent. In fact, Mendelian randomization studies usually rely on independent variants, so we will mainly focus on that case.

#### 2.2.2 | Bayesian inference for Mendelian randomization

In order to construct the JAM-MR algorithm, we first outline how a fully Bayesian algorithm for Mendelian randomization can be developed. Fully Bayesian algorithms for Mendelian randomization are not common in the literature, despite some recent developments.^30^

Suppose again that individual-level data *x*_*i*_*,y*_*i*_*,G*_1*i*_*,G*_2*i*_ were available. The data can be modeled in terms of the genetic associations with the risk factor and outcome, *𝛽*_*X*_*,𝛽*_*Y*_, and the corresponding residual variances, $\sigma_{X}^{2},\sigma_{Y}^{2}$ (alternatively, one could set *𝛽*_*Y*_ = *𝜃𝛽*_*X*_ and model the causal effect parameter explicitly). The likelihood for the risk factor and outcome in such a model would have the form $p\left(y,x|G_{1},G_{2},\beta_{X},\beta_{Y},\sigma_{X}^{2},\sigma_{Y}^{2},\gamma \right)$ Since in a two-sample framework, the data (*x,G*_1_) and (*y,G*_2_) come from two independent samples, the likelihood factorizes into

(13) $$p\left(y,x|G_{1},G_{2},\beta_{X},\beta_{Y},\sigma_{X}^{2},\sigma_{Y}^{2},\gamma \right)=p\left(y|G_{2},\beta_{Y},\sigma_{Y}^{2},\gamma \right)p\left(x|G_{1},\beta_{X},\sigma_{X}^{2},\gamma \right).$$

For Bayesian inference, this likelihood needs to be combined with a set of prior distributions on the model parameters, $p\left(\beta_{X},\beta_{Y},\sigma_{X}^{2},\sigma_{Y}^{2},\gamma \right)=p(\gamma )p\left(\beta_{X},\sigma_{X}^{2}|\gamma \right)p\left(\beta_{Y},\sigma_{Y}^{2}|\gamma \right)$. This allows us to obtain the posterior distribution

$$\begin{array}{c} p\left(\beta_{X},\beta_{Y},\sigma_{X}^{2},\sigma_{Y}^{2},\gamma |y,x,G_{1},G_{2}\right)\propto p\left(y|G_{2},\beta_{Y},\sigma_{Y}^{2},\gamma \right)p\left(x|G_{1},\beta_{X},\sigma_{X}^{2},\gamma \right)p\left(\beta_{Y},\sigma_{Y}^{2}|\gamma \right)p\left(\beta_{X},\sigma_{X}^{2}|\gamma \right)p(\gamma )\\ \propto \left[p\left(x|G_{1},\beta_{X},\sigma_{X}^{2},\gamma \right)p\left(\beta_{X},\sigma_{X}^{2}|\gamma \right)p(\gamma )\right]p\left(y|G_{2},\beta_{Y},\sigma_{Y}^{2},\gamma \right)p\left(\beta_{Y},\sigma_{Y}^{2}|\gamma \right) \end{array}$$

The term in the brackets is the posterior distribution targeted by the JAM algorithm (6) while for the remaining terms some modeling assumptions about the SNP-outcome associations are necessary—for example, a normality assumption $y∼N\left(G_{2}^{T}\beta_{Y},\sigma_{Y}^{2}\right)$. If only summarized data are available, the resulting posterior must be approximated by summary statistics, similar to (9).

#### 2.2.3 | Bayesian inference with loss functions

Instead of full Bayesian inference, the JAM-MR algorithm implements a simplified version of the above procedure. To do so, it utilizes a recently proposed framework for performing Bayesian inference using loss functions.^22^ This new theoretical framework constitutes a generalization of the core Bayesian paradigm. For a dataset D parameter vector *𝜆* and prior distribution (*𝜆*), the standard Bayesian updating scheme, p(λ∣D)∝π(λ)p(D∣λ) is replaced with a loss-function update of the form

(14) $$p_{\ell }(\lambda |D)\propto \pi (\lambda )\exp^{-w\ell (D,\lambda )},$$

where ℓ(D∣λ) represents a loss function. Here, *w* is a tuning parameter that determines the influence of the loss function relative to that of the prior. If w=1 and ℓ(D,λ)=−logp(D∣λ) is the negative log-likelihood, we obtain traditional Bayesian inference.

Sometimes this new framework is implemented using both a log-likelihood and a loss function,

(15) $$p_{\ell }(\lambda |D)\propto \pi (\lambda )p(D|\lambda )\exp\{-w\ell (D,\lambda )\}$$

which is equivalent to using $\ell (D,\lambda )-\frac{1}{w}\log p(D|\lambda )$ as a loss function in (14). The specification of a loss function is application-specific. Different loss functions can be used to tailor inference towards specific objectives, or to avoid the need to specify a full likelihood for a complex model.^22^

#### 2.2.4 | JAM with a pleiotropic loss function

JAM-MR utilizes general Bayesian inference to perform variable selection for Mendelian randomization. Our new algorithm uses the Bayesian framework only for variable selection instead of performing full Bayesian inference. Its target parameter is therefore the model indicator *𝛾* and not the full set of parameters.

JAM-MR uses an implementation of the general Bayesian framework that is similar to (15), using both a “likelihood part” and a loss function. This can be motivated by comparing (15) to the posterior distribution (13). In the “likelihood part,” we model genetic associations with the risk factor using $p\left(x|G_{1},\beta_{X},\sigma_{X}^{2},\gamma \right)$ which is the likelihood for the original JAM algorithm (but not a full likelihood for Mendelian randomization). Its purpose is to prioritize the selection of variants strongly associated with the risk factor. The loss function models genetic associations with the outcome and replaces $p\left(y|G_{2},\beta_{Y},\sigma_{Y}^{2},\gamma \right)$ in (13). We use the loss function to downweight variants which exhibit pleiotropic effects on the outcome. The tuning parameter| *w* balances the impact of the pleiotropic loss on model selection relative to that of the JAM likelihood and the prior.

Prior distributions for the SNP-risk factor association parameters $\beta_{X},\sigma_{X}^{2}$ and the model coefficient *𝛾* can be specified as in JAM. A marginalization procedure similar to that used in JAM reduces the “likelihood part” to the marginal likelihood (9).

We now discuss how to specify the loss function. It is common in the Mendelian randomization literature to use some measure of heterogeneity between univariate causal effect estimates as a proxy for pleiotropic behavior.^7,10^ The intuition is that genetic variants which are valid instruments yield the same univariate causal effect estimates, up to some random variation. On the other hand, estimates based on pleiotropic variants can exhibit systematic differences, especially if the variants operate on different causal pathways towards the outcome, because the estimated causal effects depend on the strength and direction of the pleiotropic G-Y association. This suggests that our loss function should up weight modelswith homogeneous univariate causal effect estimates, as such models are likely to contain valid instruments, and downweight modelswithheterogeneousestimates,asatleastsomeofthegeneticvariantscontainedinthemarelikelytobepleiotropic.

Consequently, in order to penalize pleiotropic models, we need a loss function that measures the heterogeneity of univariate estimates. A simple option is

(16) $$\ell_{1}(\hat{\theta },\gamma )=\frac{1}{P_{\gamma }-1}\sum_{j\in R_{\gamma }}{\left({\hat{\theta }}_{j}-{\hat{\theta }}_{\gamma ,\text{IVW}}\right)}^{2},$$

Where

(17) $${\hat{\theta }}_{\gamma ,\text{IVW}}=\frac{\sum_{j\in R_{\gamma }}{\hat{\theta }}_{j}{\hat{s}}_{\theta j}^{-2}}{\sum_{j\in R_{\gamma }}{\hat{s}}_{\theta j}^{-2}},$$

(18) $${\hat{s}}_{\gamma ,\text{IVW}}^{2}=\text{ s}.\text{e}.\;{\left({\hat{\theta }}_{\gamma ,\text{IVW}}\right)}^{2}=\frac{1}{\sum_{j\in R_{\gamma }}{\hat{s}}_{\theta j}^{-2}}.$$

is the inverse-variance-weighted (IVW) causal effect estimate based on variants in the set *R*_*𝛾*_. The loss function (16) is the sum of squared differences of SNP-specific estimates ${\hat{\theta }}_{j}$ from the causal effect estimate ${\hat{\theta }}_{\gamma ,IVW}$ computed using all SNPs in the current model. If a model contains heterogeneous variants, some of these differences will be large, the sum-of-squares will be large and the term exp{−wℓ(D,θ)} in (15) will be small; therefore, heterogeneous models are downweighted.

Note that the loss function (16) is not defined for the null model and models containing only one genetic variant. Such models carry no evidence as to whether the variant included is valid or pleiotropic. Therefore, we ignore these models and restrict JAM-MR to only consider models with at least two genetic variants. Since JAM-MR works with a single risk factor, our assumption that there are at least two genetic variants is similar to overidentification assumptions used in econometrics to test for instrument validity. In both cases, the assumption is needed because a single instrument would provide insufficient information to assess violations of the instrumental variable assumptions. However, JAM-MR and overidentification tests use the assumption in different ways. For example, in Sargan’s J test, the number of instruments minus the number of endogenous variables determines the degrees of freedom of the test’s chi-squared distribution. On the other hand, the process of fitting JAM-MR is not directly affected by the number of genetic variants, provided there are two or more variants.

An alternative loss function can be obtained by weighting the individual causal effect estimates in (16) by the inverse of their squared SEs,

(19) $$\ell_{2}(\hat{\theta },\gamma )=\sum_{j\in R_{\gamma }}{\left({\hat{\theta }}_{j}-{\hat{\theta }}_{\gamma ,\text{IVW}}\right)}^{2}{\hat{s}}_{\theta j}^{-2}.$$

This second loss function has the advantage that it also models uncertainty in the univariate ratio estimates ${\hat{\theta }}_{j}$ through its dependence on the SEs ${\hat{s}}_{\theta j}^{2}$. A similar function has been used^10^ to obtain a pleiotropy-robust exhaustive-search model averaging procedure for Mendelian randomization. In the simulations and the real-data application of this article, we have used the weighted loss function (19).

Combining the general Bayesian inference framework (15), the JAM likelihood (9) and the loss function (19) and using the summary-level data $\left({\hat{\beta }}_{X},{\hat{\sigma }}_{X}^{2},\hat{\theta },{\hat{s}}_{\theta },G_{\text{ref }}\right)$ instead of the individual-level data (*y,x,g*_1_*,G*_2_), the Bayesian loss-posterior for JAM-MR’s variable selection is

(20) $$p_{\ell }\left(\gamma |{\hat{\beta }}_{X},{\hat{\sigma }}_{X}^{2},\hat{\theta },{\hat{s}}_{\theta },G_{\text{ref }}\right)\propto \frac{B\left(a_{\omega }+P_{\gamma },b_{\omega }+P-P_{\gamma }\right)}{B\left(a_{\omega },b_{\omega }\right)}{\left(\tau +1\right)}^{-\frac{P_{\gamma }}{2}}{\left(2b_{\sigma }+S(\gamma )\right)}^{-a_{\sigma }-\frac{N_{1}}{2}}\times \exp\left\{-w\sum_{j\in R_{\gamma }}{\left({\hat{\theta }}_{j}-{\hat{\theta }}_{\gamma ,\text{IVw}}\right)}^{2}{\hat{s}}_{\theta j}^{-2}\right\},$$

where *S*(*𝛾*) can be computed from (11). Note that this formula still depends on the tuning parameter *w*; the specification of a value for *w* will be discussed in Section 2.2.6. Analytic calculation of the loss-posterior is infeasible, since it depends on a normalizing constant whose evaluation would require us to sum over all 2^*P*^ possible models. However, the loss-posterior can easily be evaluated up to a normalizing constant and hence it can be used as the target distribution for JAM-MR’s reversible-jump MCMC algorithm. This stochastic search procedure scales well to large datasets containing hundreds of genetic variants, is quite flexible and can explore large parts of the model space.

Since we are using heterogeneity as a proxy for pleiotropic behavior, JAM-MR implicitly makes a plurality assumption: the algorithm targets the largest set of genetic variants with homogeneous (ie, asymptotically equal) univariate causal effect estimates, and it is assumed that this set corresponds to the valid instruments. Similar assumptions have been made by other methods.^7,10,15^

#### 2.2.5 | Causal effect estimation using JAM-MR

The JAM-MR posterior probabilities can subsequently be used to obtain an overall estimate of the causal effect of interest, according to a model averaging procedure. The use of model averaging enables quantification of uncertainty related to the choice of instruments and allows many genetic variants to have small contributions to the overall causal effect estimate.

For each model, the algorithm computes a model-specific causal effect estimate ${\hat{\theta }}_{\gamma }$ and SE ${\hat{s}}_{\gamma }$. These are then combined into a single estimator,

(21) $${\hat{\theta }}_{\text{JAM}-\text{MR}}=\sum_{\gamma }p(\gamma |x){\hat{\theta }}_{\gamma },$$

with variance

(22) $${\hat{s}}_{\text{JAM}-\text{MR}}^{2}=\sum_{\gamma }p(\gamma |x){\hat{s}}_{\gamma }^{2}+\sum_{\gamma }p(\gamma |x){\hat{\theta }}_{\gamma }\left({\hat{\theta }}_{\gamma }-{\hat{\theta }}_{\text{JAM}-\text{MR}}\right),$$

where the summation is over all models *𝛾* assigned positive posterior probability by the variable selection procedure. Equation (22) can be derived as an approximation to the posterior variance Var(θ∣D), by expressing it in terms of the model-specific posterior moments E(θ∣D,γ),Var(θ∣D,γ) and approximating these by ${\hat{\theta }}_{\gamma },{\hat{s}}_{\gamma }^{2}$.

A straightforward choice for the model-specific estimates would be the IVW estimate (17). However, we have observed empirically that this choice often results in underestimation of the overall causal SE and a more elaborate modeling assumption may be needed. Because JAM-MR uses the heterogeneity of ratio estimates in order to model pleiotropic behavior, it will tend to favor sets of variants with homogeneous causal effect estimates and consequently underestimate the overall heterogeneity. This manifests as overly precise SEs, as estimated from the naive IVW formula. A relevant example is given in the supplement.

To better model this behavior and attenuate the overprecision, we modify the IVW estimates as follows. The standard IVW formula can be motivated as the maximum likelihood estimate obtained from the heteroskedastic linear regression model ${\hat{\theta }}_{j}∼N\left(\theta ,{\hat{s}}_{\theta j}^{2}\right)$. To account for the presence of moderately pleiotropic variants, which may have been inadvertently included in JAM-MR’s model due to their proximity to valid instruments, it has been proposed^31^ to use a multiplicative random effects model of the form ${\hat{\theta }}_{j}∼N\left(\theta ,\phi^{2}{\hat{s}}_{\theta j}^{2}\right)$. This results in the same causal effect estimate (17) as the IVW approach, but scales its SE by *𝜙*. The overdispersion parameter *𝜙* can be estimated using weighted linear regression.^31^ On the other hand, to model the potential downweighting of valid genetic variants with ratio estimates that may seem outlying due to random variation, we modify the random-effects model further and use a truncated normal distribution for the univariate causal effects:

(23) $${\hat{\theta }}_{j}∼TN\left(\theta ,\phi^{2}{\hat{s}}_{\theta j}^{2},l,u\right),$$

where *l*, *u* denote the lower and upper truncation points. Using (23) we can obtain a model-specific likelihood

(24) $$\ell \left(\theta ,\phi^{2}\right)=\prod_{j\in R_{\gamma }}f_{TN}\left({\hat{\theta }}_{j};\theta ,\phi^{2}{\hat{s}}_{\theta j}^{2},l,u\right)=\prod_{j\in R_{r}}\frac{1}{\phi {\hat{s}}_{\theta j}}\frac{\exp\left\{-\frac{{\left({\hat{\theta }}_{j}-\theta \right)}^{2}}{2\phi^{2}s_{sj}^{2}}\right\}}{\text{Φ}\left(\frac{u-\theta }{\phi {\hat{s}}_{\theta j}}\right)-\text{Φ}\left(\frac{l-\theta }{\phi {\hat{s}}_{\theta j}}\right)},$$

where Φ denotes the cumulative distribution function of a *N*(0,1) random variable. The likelihood (24) can then be maximized numerically to obtain a model-specific estimate ${\hat{\theta }}_{\gamma }$ of the causal effect. To simplify the optimization problem, we fix the left truncation point *l* to the value $\hat{l}=\underset{k\notin \gamma }{\max}\left\{{\hat{\theta }}_{k}:{\hat{\theta }}_{k}<\underset{j\in R_{\gamma }}{\min}{\hat{\theta }}_{j}\right\}$. This is the equivalent of truncating at the largest ratio estimate ${\hat{\theta }}_{k}$ of a SNP not in *R*_*𝛾*_ that is smaller than all the ratio estimates of SNPs in *𝛾*. If no such ${\hat{\theta }}_{k}$ exists, we do not truncate to the left $\left(\hat{l}=-\infty \right)$. Likewise, we fix the right truncation point at $\hat{u}=\min_{k\notin \gamma }\left\{{\hat{\theta }}_{k}:{\hat{\theta }}_{k}>\max_{j\in R_{\gamma }}{\hat{\theta }}_{j}\right\}$ the smallest ratio estimate of a SNP not in *R*_*𝛾*_ that is still larger than the ratio estimates of all SNPs in *𝛾* (or $\hat{u}=\infty ,$ if no such ${\hat{\theta }}_{k}$ exists).

Optimization of (24) over (*𝜃,*^2^) can be performed using gradient-based methods, since the derivatives of the likelihood (24) are straightforward to compute. To estimate the causal SE, we rely on frequentist asymptotic arguments and compute the sample Fisher information matrix,

(25) $$I\left(\theta ,\phi^{2}\right)=E\left[-\frac{\partial^{2}}{\partial {\left(\theta ,\phi^{2}\right)}^{2}}\log\ell \left(\theta ,\phi^{2}\right)\right]$$

evaluated at the maximum likelihood estimates (*𝜃,^ 𝜙̂*^2^). The maximum likelihood estimate for *𝜃* and its SE are then used as the model-specific estimates in (21), (22), from which to obtain the overall causal effect estimate.

This truncated multiplicative random-effects model has been used in the simulations and the real-data application of the paper. A comparison between JAM-MR implementations with fixed-effects IVW, random-effects IVW and the truncated approach for model-specific estimates is provided in supplementary material.

Regardless of how the SEs are computed, we note that this part of our algorithm is not Bayesian: the process of obtaining model-specific causal effect estimates relies on maximum likelihood estimation. Therefore, the interpretation ofJAM-MR’s causal effect estimate should be viewed under this perspective. Likewise, we refer to high-probability regions for *𝜃* as confidence intervals, rather than credible intervals.

#### 2.2.6 | Estimation of the tuning parameter *w*

The tuning parameter *w* plays a crucial role in JAM-MR’s variable selection. Tuning *w* is subject to a bias-variance trade-off. For relatively small values, the pleiotropic loss function has limited effect on the variable selection procedure and JAM-MR tends to favor larger models. These models may still include some pleiotropic variants, and the resulting causal effect estimates may exhibit bias. On the other hand, with a large value of *w* the algorithm favors models that contain no pleiotropic variants but may also ignore some of the valid instruments. In this case JAM-MR yields unbiased causal effect estimates, but these estimates may have large SEs.

Setting *w* = 0 is equivalent to the standard JAM algorithm with no pleiotropy adjustment; the algorithm is similar to a simple IVW estimator except it downweights genetic variants that are weakly associated with the risk factor. The other extreme case is to assign a very large value to *w*. In this case the algorithm becomes very selective and prioritizes models containing only two genetic variants. We refer to the supplementary material for more details on the algorithm’s behavior with large *w*.

In order to balance between these two extremes, JAM-MR implements a grid search to select the value of the tuning parameter. A range of candidate values is specified and the stochastic search is run for each value separately. Candidate values for the grid search should scale according to the sample size *N*_1_, since the value of the likelihood also scales according to *N*_1_. By default, JAM-MR uses a grid with 25 points, spaced equally on the logarithmic scale between *w*_1_ = 0.01*N*_1_ and *w*_25_ = 100*N*_1_ (ie, log*w*_1_, …,log*w*_25_ are equally spaced), plus an implementation for *w*_0_ = 0.

Among the candidate values for *w*, the algorithm then selects the one for which the smallest causal SE (22) is obtained. Intuitively, the collection of genetic variants that attains the smallest SE should correspond to the set of valid instruments, as both the inclusion of a pleiotropic variant and the removal of a valid SNP from that model will increase the causal SE. In our simulations, we have found this criterion to offer a reasonable compromise in the bias-variance trade-off that underpins the selection of a value for the tuning parameter.

## 3 | SIMULATION STUDY

### 3.1 | Simulation setting

We conducted a simulation study in order to assess the performance of the JAM-MR algorithm and compare it to established approaches for Mendelian randomization. The Mendelian randomization model that we used for the simulations was

(26) $$U_{i}=\sum_{j=1}^{P}\alpha_{j}G_{ij}+\epsilon_{Ui}$$

(27) $$X_{i}=\sum_{j=1}^{P}\beta_{Xj}G_{ij}+c_{X}U_{i}+\epsilon_{Xi}$$

(28) $$Y_{i}=\theta X_{i}+\sum_{j=1}^{P}\delta_{j}G_{ij}+c_{Y}U_{i}+\epsilon_{Yi}$$

Where $\epsilon_{Ui}∼N\left(0,\sigma_{\epsilon_{U}}^{2}\right),\epsilon_{Xi}∼N\left(0,\sigma_{\epsilon_{X}}^{2}\right),\epsilon_{Yi}∼N\left(0,\sigma_{\epsilon_{Y}}^{2}\right)$ independently of each other. The effects of genetic variant *G*_*j*_ on *U,X,Y* are denoted by *𝛼*_*j*_*,𝛽*_*j*_*,𝛿*_*j*_, respectively. The parameters *𝛿*_*j*_ represent the direct effects of genetic instruments on the outcome. The parameters *𝛼*_*j*_ represent effects mediated by confounders, which we use to model violations of the InSIDE assumption (“instrument strength is independent of direct effects”). For genetic variants that are valid instruments, *𝛿*_*j*_ = *𝛼*_*j*_ = 0.

Our simulations were conducted using the statistical software R. To inform the simulations, we used sample sizes and numbers of genetic variants similar to those in the real-data application presented in the following section. Specifically, we used a two-sample Mendelian randomization setting and generated two sets of individual-level data for *N*_1_ = *N*_2_ = 300 000 individuals and *P* = 400 independent genetic variants. These sample sizes are in line with recent Mendelian randomization analyses using composite risk factors. Of the 400 genetic variants, *P*_1_ = 120 (30%) were simulated as pleiotropic and *P* − *P*_1_ = 280 were simulated as valid instruments.

Genetic variants *g*_*ij*_ were generated from a Binomial(2*,f*_*j*_), with effect allele frequencies *f*_*j*_ ∼ *U*(0.1,0.9). The genetic effects on the risk factor were simulated as *𝛽*_*Xj*_ ∼ 0.5 + *N*(0,0.5^2^), which we empirically observed to yield a realistic pattern of genome-wide significant *p*-values, the majority of which were between 10^−7^ and 10^−50^. The error term variances were set equal, $\sigma_{\epsilon_{U}}^{2}=\sigma_{\epsilon_{X}}^{2}=\sigma_{\epsilon_{Y}}^{2}=\sigma_{e}^{2}$ and the value of $\sigma_{\epsilon }^{2}$ was specified so that 10% of the variation in the risk factor is explained by the genetic variants. We also set *c*_*X*_ = *c*_*Y*_ = 1.

The pleiotropic effect parameters *𝛼*_*j*_, *𝛿*_*j*_ were set equal to zero for all the valid instruments. For invalid instruments, we generated three simulation scenarios. In the first scenario (balanced pleiotropy), we let *𝛼*_*j*_ = 0 and *𝛿*_*j*_ ∼ ±*N*(0.7,0.2^2^), setting the sign of each *𝛿*_*j*_ to be positive or negative with equal probability. In the second scenario (directional pleiotropy, InSIDE assumption satisfied) we also set *𝛼*_*j*_ = 0 but the direct effect parameters *𝛿*_*j*_ were restricted to be positive, *𝛿*_*j*_ ∼ *N*(0.7,0.2^2^). Finally, in the third simulation scenario (directional pleiotropy, InSIDE violated), the SNP-confounder effects were also randomly generated, *𝛼*_*j*_ ∼ *N*(0.4,0.2^2^). The direct effects *𝛿*_*j*_ were generated as in the second scenario. The three simulation scenarios are summarized in Table 1. Plots of univariate *p*-values for SNP-trait associations and univariate causal effect estimates for our simulations are provided in the supplementary material.

For each scenario, we implemented two sets of simulations, one with a null (*𝜃* = 0) and one with a positive (*𝜃* = 0.3) causal effect. This resulted in a total of six simulation settings. Each setting was replicated 1000 times. In each replication, we generated the individual-level data and then performed univariate linear regression between each genetic variant and the risk factor and outcome to obtain GWAS summary statistics. Using these summary statistics, we then implemented JAM-MR as well as a range of already existing algorithms for Mendelian randomization. For comparison purposes, we also computed “oracle” IVW causal effect estimates using only the valid instruments. For more details on how JAM-MR and the competing methods were implemented in our simulation, we refer to the supplementary material.

### 3.2 | Competing methods

To assess the performance of the JAM-MR algorithm, we compared it against the following established methods for Mendelian randomization:

- Standard IVW estimation.
- MR-Egger regression.^5^
- Median estimation.^6^
- Mode-based estimation.^7^
- A Lasso-type estimator.^9^
- MR-Presso.^11^
- MR-Raps.^12^
- Contamination mixture.^15^

The simulations were conducted in the statistical software R. The code we used to implement JAM-MR is publicly available as part of the (GitHub) R package R2BGLiMS. An outline of each of the competing methods and their implementation in R is provided in the supplement. For further details the reader is referred to the relevant citations.

### 3.3 | Simulation results

The results of this simulation experiment are reported in Table 2. We report average causal effect estimates, estimated SEs and root mean squared errors. SEs for the contamination mixture are not reported because the method does not compute SEs and directly obtains a confidence region (which is not necessarily a single interval) instead. In the case of a null causal effect, we also report Type I error rates (calculated as the empirical power to reject the null causal hypothesis) at a 95% significance level, while for simulations with a positive causal effect we report the empirical coverage of 95% confidence intervals. We do not report the power to reject the null hypothesis when it is not true, because all methods were able to do so in almost all the simulation replications; this was due to the large sample sizes that we used, in combination with *𝜃* = 0.3 being a rather strong effect that most methods could easily identify and the fact that pleiotropic bias in our simulations was in the direction away from the null.

In the first simulation (“balanced pleiotropy”), all Mendelian randomization methods provided nearly unbiased estimates of the causal effect of interest, especially for *𝜃* = 0. This was the case even for the standard inverse-variance weighted method, which does not perform pleiotropy adjustments (the IVW was implemented with a random-effects adjustment, hence the fairly accurate SE estimates). The JAM-MR implementation attained SEs close to those obtained by the oracle IVW and had one of the smallest mean squared errors among the competing approaches. In terms of calibration of confidence intervals, JAM-MR exhibited a slight inflation of Type I error rates for *𝜃* = 0 and a moderate reduction in coverage for *𝜃* = 0.3. Most of the competing methods attained nominal Type I error rates for *𝜃* = 0, but the Lasso, MR-Presso and the contamination mixture performed worse than JAM-MR. The contamination mixture in particular exhibited significant inflation; this was due to the fact that the method’s default values for its tuning parameters were not suitable for modeling the simulated datasets. For *𝜃* = 0.3, all methods except the mode, MR-Raps and possibly IVW exhibited a reduction in coverage. The performance of most methods was slightly worse in this case compared with a null causal effect. We conjecture that this might have been due to mild effects of weak instrument bias. Instrument strength in our simulations was calibrated using heuristic arguments based on our real-data application, as discussed earlier. The value of the F statistic in our simulations was well above the threshold of 10 that indicates weak instrument bias, ranging between 50 and 100 in most scenarios. Nevertheless, it is possible that a small amount of bias might have remained, affecting causal effect estimates in the third significant digit and causing the mild undercoverage observed by many methods. Weak instrument bias is known to act towards the null in two-sample Mendelian randomization, which would explain why most methods performed better for *𝜃* = 0, and would also explain why undercoverage is observed even for the oracle IVW procedure.

In the second scenario (“directional pleiotropy, InSIDE assumption satisfied”), there were larger deviations in the performance of the various methods. Bias in causal effect estimates was observed for the majority of methods, with mode-based estimation, the contamination mixture and JAM-MR being the least biased algorithms on average. Among these three approaches, JAM-MR and the contamination mixture attained smaller mean squared errors. The SEs computed using JAM-MR were smaller than those of the mode and again close to those of the oracle estimator. On the other hand, the mode-based approach offered better calibration of Type I error rates and coverage of confidence intervals; in fact, the method was conservative and attained Type I error rates below the 5% threshold. Both JAM-MR and the contamination mixture exhibited an inflation in Type I error rates, although the issue was less pronounced for JAM-MR. A similar pattern of results was observed in simulation scenario 3 (“directional pleiotropy, InSIDE assumption violated”).

Among the other methods, MR-Egger returned fairly accurate causal effect estimates and well-calibrated confidence intervals in scenario 2, but had a significantly higher SE compared with all the other approaches. The lack of power associated with MR-Egger is well-known in the Mendelian randomization literature.^16^ MR-Egger also depends on the InSIDE assumption, therefore its performance was poor in scenario 3 where this assumption was violated. The remaining methods were subject to different degrees of bias in both directional pleiotropy scenarios with the Lasso approach being the least biased. This inaccuracy also gave rise to large Type I error rates.

The Type I error rates observed for some of the competing methods are rather extreme. These error rates were mainly due to biases in causal effect estimates. Our simulation was rather challenging, as it contained a large number of pleiotropic variants with similar univariate causal effect estimates and this was difficult for some of the methods to tackle. For example, the MR-Presso method relies on outlier removal and can be expected to struggle in simulations where many genetic variants exhibit similar pleiotropic effects. We should mention that many of the existing methods would perform quite well in different simulation scenarios, and some of them (such as the median and MR-Raps) come with theoretical guarantees of good asymptotic performance. Hence, their poor behavior here should not be taken as a general-purpose criticism towards these methods.

In comparison, JAM-MR offered causal effect estimates with low bias even in challenging simulation scenarios. On the other hand, the algorithm’s suboptimal calibration of confidence intervals is a concern and merits some discussion. Upon inspection, the algorithm’s estimated SEs are smaller than the empirical SD of estimates across multiple iterations; this happens both for fixed *w* and for the grid search version of the algorithm. An explanation for this could be that our heuristic multiplicative truncated random effects procedure, though more efficient than standard IVW, is still unable to perfectly model the uncertainty in model-specific estimates. Alternatively, the undercoverage could be an indication that JAM-MR’s reversible-jump MCMC algorithm exhibits insufficient mixing. In each iteration, our algorithm proposes moves that modify the selected model by only a single SNP, and can find it difficult to move between two models containing completely different sets of SNPs, when such models have high posterior probability; focusing on one of the two models would result in overprecise estimates. Regardless of the reason, the below-nominal coverage of the algorithm can be a concern, but we believe that JAM-MR still possesses some utility as a sensitivity tool, by virtue of offering accurate causal effect estimation, as well as additional diagnostics for pleiotropy such as posterior inclusion probabilities for each variant and plots of causal effect estimates per value of *w*, as presented below.

In the supplement we consider simulations with different GWAS sample sizes, different numbers of genetic variants, a varying proportion of genetic variation in the risk factor that is explained by the genetic variants, different proportions of pleiotropic variants and a “mild directional pleiotropy” simulation where not all invalid instruments have pleiotropic effects in the same direction. In the majority of simulations, JAM-MR was able to consistently estimate the causal effect of interest, at the cost of a small reduction in coverage of confidence intervals. Of note was a small deterioration in the algorithm’s performance when both the GWAS sample sizes and the proportion of genetic variation in the risk factor were small. The mode and the contamination mixture remained the best competitors throughout these simulations. We also implemented simulations where either the confounder, risk factor or outcome were not generated from a normal distribution. These simulations suggested that pleiotropic bias was a bigger concern for most methods than violations of normality assumptions.

**FIGURE 2:**
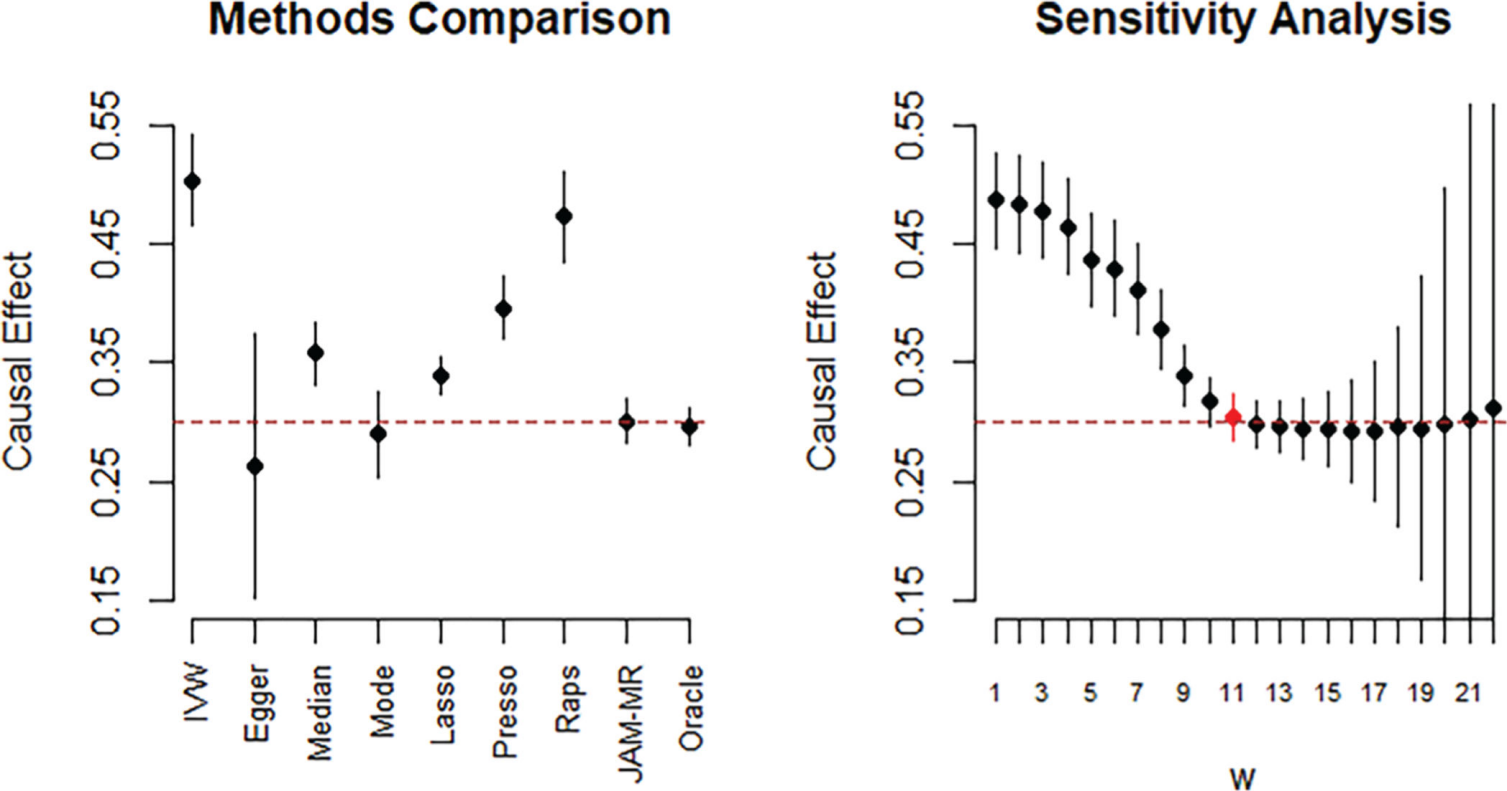
Simulation scenario 2: Directional pleiotropy, InSIDE satisfied (*𝜃* = 0.3). Causal effect estimates and 95% confidence intervals for various Mendelian randomization algorithms (left) and JAM-MR with a range of *w* values (right). The dashed line at 0.3 represents the true causal effect [Colour figure can be viewed at wileyonlinelibrary.com]

In the left-hand panel of Figure 2 we visualize the causal effect estimates obtained from the various methods for the directional pleiotropy simulation with the InSIDE assumption satisfied (scenario 2) and *𝜃* = 0.3. In addition, in the right-hand panel of Figure 2, we have plotted causal effect estimates and confidence intervals obtained by running JAM-MR with various values of *w*. The plot illustrates how the algorithm can provide insight into the pattern of pleiotropy in the data. Causal effect estimates for small values of *w* were much larger than those for moderate and large values, indicating that pleiotropic variants are present in the dataset and cause upwards bias if not accounted for. As we increase *w* towards the minimum-standard-error value, plotted in red, causal effect estimates become unbiased. However, for large values of *w* the estimates become more variable and eventually unstable, as we discuss in the supplementary material. This is because the pleiotropic loss function removes most of the valid SNPs and causal effect estimation is based on only a small number of genetic variants, increasing SEs and reducing the power to detect a causal association.

**FIGURE 3:**
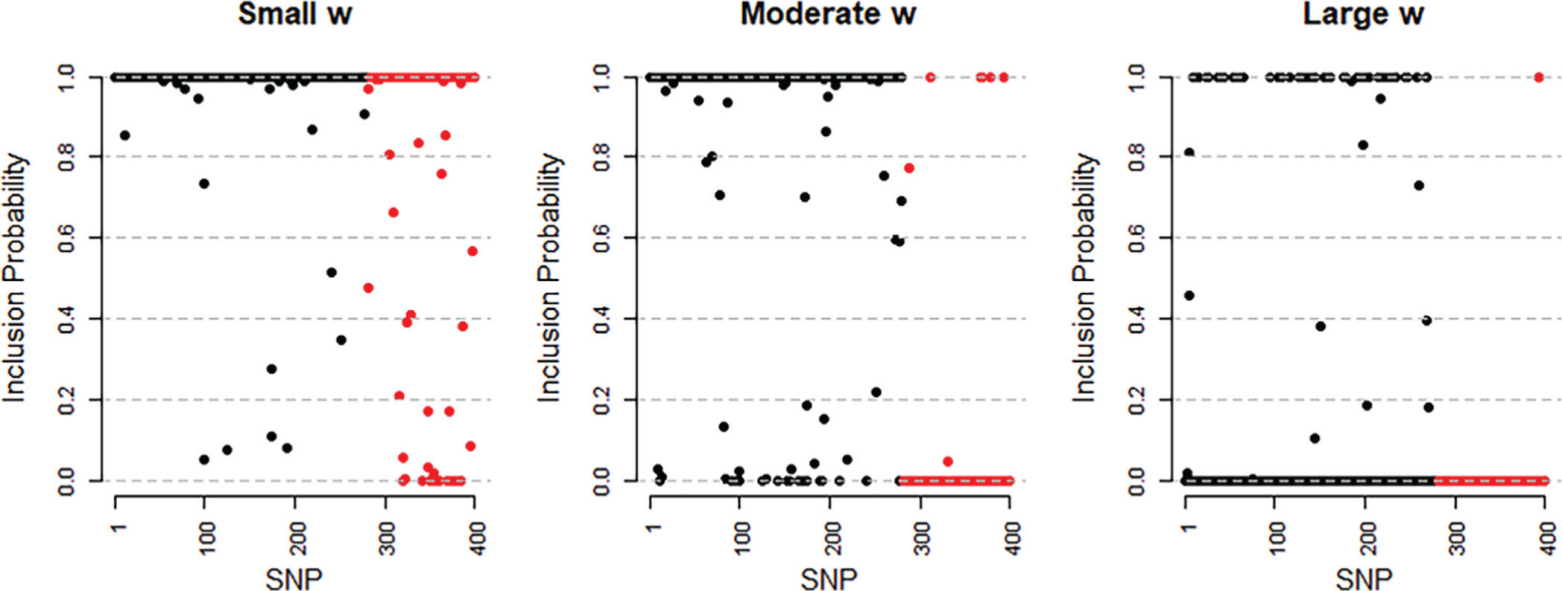
Manhattan plots illustrating the posterior inclusion probabilities assigned to each SNP by the JAM-MR algorithm with small (left), moderate (center), and large (right) values of *w*, for a single implementation of simulation scenario 2 with *𝜃* = 0.3 [Colour figure can be viewed at wileyonlinelibrary.com]

The JAM-MR algorithm returns posterior inclusion probabilities for each genetic variant, which can be plotted in a Manhattan plot. In Figure 3, we have plotted inclusion probabilities for a single implementation of the directional pleiotropy simulation scenario 2 with *𝜃* = 0.3. We used three JAM-MR runs with a small (*w* ≈ 0.03*N*_1_), a moderate (*w* ≈ 0.4*N*_1_) and a large (*w* ≈ 15*N*_1_) value for the tuning parameter. Pleiotropic variants are colored red. The plot illustrates that our heterogeneity penalization procedure makes accurate selection of the valid instruments, when properly tuned (center panel). When a small value of *w* is used (left panel), the algorithm may retain some pleiotropic SNPs in the analysis. On the other hand, when *w* is large (right panel), the algorithm will correctly downweight pleiotropic variants but will also downweight some of the valid SNPs.

In our simulations, we used a 51-point grid search in order to tune the JAM-MR algorithm. In practice, it may be unnecessary to use such a large grid - often, even a grid as small as 5 to 10 values will provide a reasonable fit. To verify that, we compared the causal effect estimates obtained from JAM-MR using a 51-point grid search to those obtained using a smaller grid with only nine points, nested within the 51-point grid. The resulting causal effect estimates were quite similar for all simulation scenarios. In particular, the mean absolute difference in causal effect estimates obtained by the two grid-search procedures across all our simulations was 0.0029, and the mean absolute difference in SE estimates was 0.00047.

Overall, the JAM-MR algorithm was able to identify the causal effect of interest with reasonable accuracy in all our simulations, yielded SEs close to those obtained by the oracle IVW procedure, and fairly small root mean squared errors. This came at the expense of a slight inflation in its Type I error rates across most simulation scenarios. The most promising competing approaches were mode-based estimation and the contamination mixture. JAM-MR exhibited smaller mean squared errors than the mode, which produced better-calibrated confidence intervals in exchange. The contamination mixture yielded similar mean squared errors as JAM-MR but with a slightly larger Type I error rate inflation.

## 4 | APPLICATION: CAUSAL EFFECT OF BLOOD PRESSURE ON CHD RISK

### 4.1 | Blood pressure traits and associated genetic variants

We now illustrate the use of the JAM-MR algorithm in a real-data application. We conduct a Mendelian randomization analysis to assess the effect of blood pressure on the risk of CHD. This application has been studied in the past^5,14,32^ and it is generally accepted that high blood pressure has an increasing effect on the risk of suffering from CHD, despite the fact that a previous Mendelian randomization analysis^5^ did not identify a causal relationship.

Our analysis is novel not in the question it aims to answer but in the data sources it uses. We utilized a recently published meta-GWAS study^33^ which identified hundreds of genetic variants associated with blood pressure. The authors meta-analyzed data from the International Consortium for Blood Pressure (ICBP), the UK BioBank, the US Million Veterans Project, and the Estonian Genome Center Biobank. In total, a sample of approximately 1 million individuals of European descent was analyzed. The study confirmed previously reported findings about 258 known and 92 reported but not validated genetic variants associated with blood pressure. It also identified a total of 535 novel associations.

We used two blood pressure traits for our analysis, namely, systolic and diastolic blood pressure. For each trait, we used all 258 genetic variants with an already established relation to blood pressure, as well as any of the reported-but-not-validated and novel variants reported to be associated with that trait. Among the novel findings, we excluded from consideration variants which were associated with blood pressure in the “discovery” GWAS but not in the “replication” GWAS.^33^ This resulted in a total of *P*_1_ = 395 genetic variants for systolic and *P*_2_ = 391 genetic variants for diastolic blood pressure; our analysis was therefore based on larger numbers of genetic variants than previous Mendelian randomization investigations.

Genetic associations with systolic and diastolic blood pressure were obtained from the supplementary tables of Evangelou et al.^33^ We used estimates based on the ICBP dataset of *N*_1_ = 299 024 individuals, as this was the only dataset for which genetic associations were reported for all variants. Since the genetic associations with blood pressure were replicated in independent datasets,^33^ winner’s curse bias is unlikely to have had a serious effect in our analysis. For the selected variants, we obtained genetic associations with CHD risk from the CARDIoGRAMplusC4D Consortium,^34^ based on a sample of *N*_2_ = 184 305 individuals. The variants were mostly independent, as a result of LD pruning.^33^

### 4.2 | Mendelian randomization analysis

**FIGURE 4:**
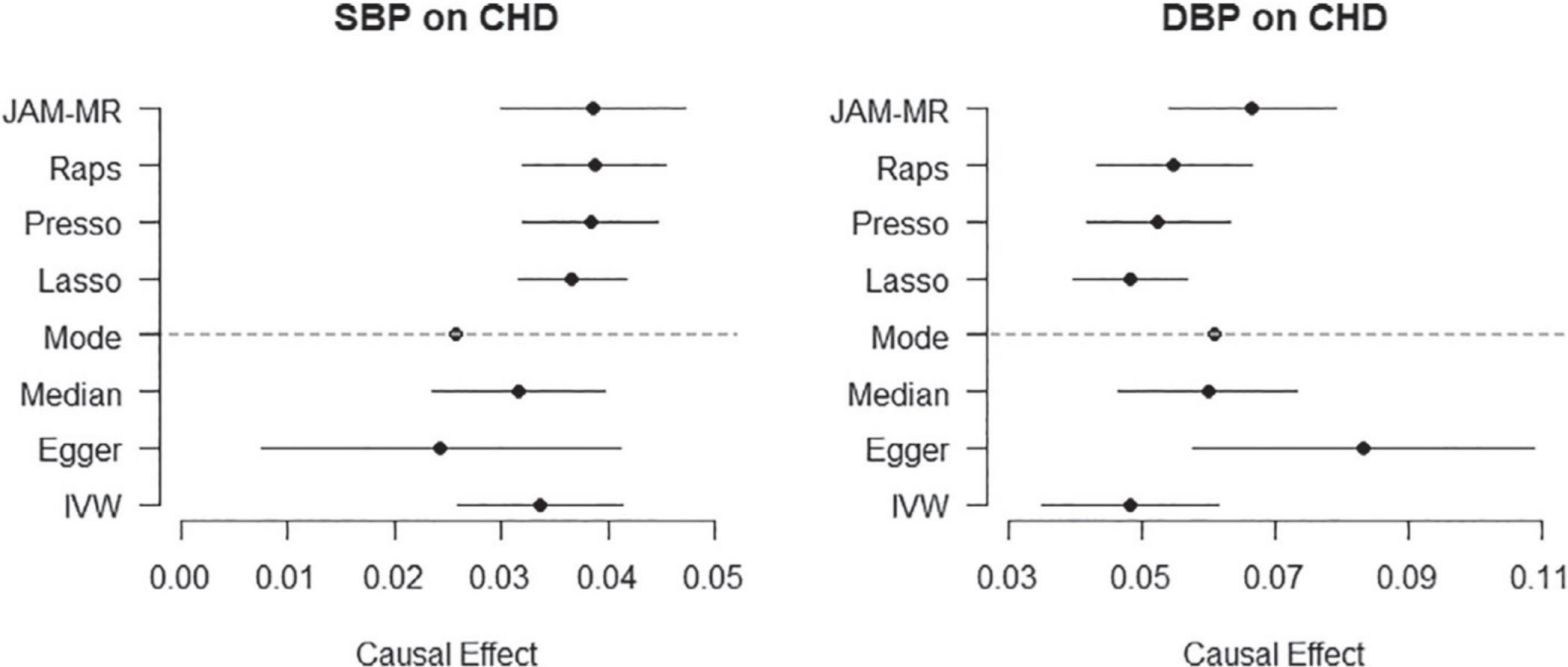
Log-odds ratios of increase in coronary heart disease risk per 1 mmHg increase in systolic and diastolic blood pressure. Point estimates and 95% confidence intervals for various Mendelian randomization methods

We implemented JAM-MR and the competing Mendelian randomization methods to identify the causal effect of systolic and diastolic blood pressure on CHD risk. The results are listed in Table 3, where we report the estimated causal effect (log-odds ratio of increase in CHD risk per mmHg increase in blood pressure measurement) and its 95% confidence interval for each of the two traits and each Mendelian randomization method. Confidence intervals for the JAM-MR algorithm were computed using the truncated random-effects approach. A graphical illustration of the results is provided in Figure 4. Tables of summary statistics, JAM-MR inclusion probabilities for each SNP and corresponding Manhattan plots can be found in the supplementary material.

The reported effects from the various methods confirm that increased blood pressure, both systolic and diastolic, has a deleterious effect on CHD risk. For systolic blood pressure, the causal effect reported by the various methods was in the region of 0.024 to 0.040, corresponding to an odds ratio of *e*^0.24^ = 1.024 to *e*^0.4^ = 1.041. All methods were able to reject the null causal hypothesis at a 95% significance threshold, except the mode-based estimator whose SE was unusually large. JAM-MR estimates were very close to those reported by other methods. The minimum causal SE criterion suggested using an implementation with *w* ≈ 0.5*N*_1_, for which the corresponding JAM-MR log-odds ratio estimate was 0.039 (95% confidence interval: (0.030,0.047)).

For diastolic blood pressure, most of the established Mendelian randomization methods reported causal effect estimates between 0.048 and 0.061, corresponding to odds ratios between 1.049 and 1.063. The JAM-MR estimates were slightly larger, yielding a log-odds ratio of 0.067 (95% confidence interval (0.054,0.079)) and an effect estimate of *e*^0.067^ = 1.069. These were attained for *w* ≈ 0.29*N*_1_. This difference is mainly due to JAM-MR downweighting genetic variants that are weak instruments; the estimate of 0.067 is close to what would be obtained by running the competing methods using only the 258 genetic variants with established associations with blood pressure. Once again, all methods were able to reject the null causal hypothesis at a 95% level, except the mode-based estimator.

### 4.3 | Interpretation of results

The two JAM-MR implementations assigned posterior inclusion probabilities larger than 0.5 to 142 genetic variants for systolic and 169 variants for diastolic blood pressure. Of the 253 variants for systolic blood pressure that were assigned posterior probability lower than 0.5, 116 had univariate causal effect estimates below the overall reported value of 0.037 and 137 had univariate estimates above that value. For diastolic blood pressure, there were 222 variants assigned posterior probabilitieslowerthan0.5,121ofwhichhadsmallerand101hadlargerunivariateratioestimatesthanthereportedvalue of 0.067. Not all genetic variants were downweighted due to pleiotropy; some were assigned low posterior probabilities because their association with the two blood pressure traits were not strong enough. Nevertheless, the downweighting of genetic variants both larger and smaller than the overall causal effect estimates in almost equal numbers suggests a fairly balanced pleiotropic pattern. In line with that observation, the reported estimates in Table 3 resemble those for the “balanced pleiotropy” setting in Table 2 of our simulation study, with various methods producing similar causal effect estimates. This is especially the case for systolic blood pressure - for diastolic blood pressure, a small degree of pleiotropic bias towards the null may be present, considering that IVW yielded the smallest estimate among all methods considered. The analysis for diastolic blood pressure would be similar to a simulation design under the directional pleiotropy setting, but with weaker directionality (eg, 60% of the pleiotropic variants have risk-decreasing pleiotropic effects and 40% have risk-increasing effects). In supplementary material, we present and discuss the results obtained from such a simulation design.

**FIGURE 5:**
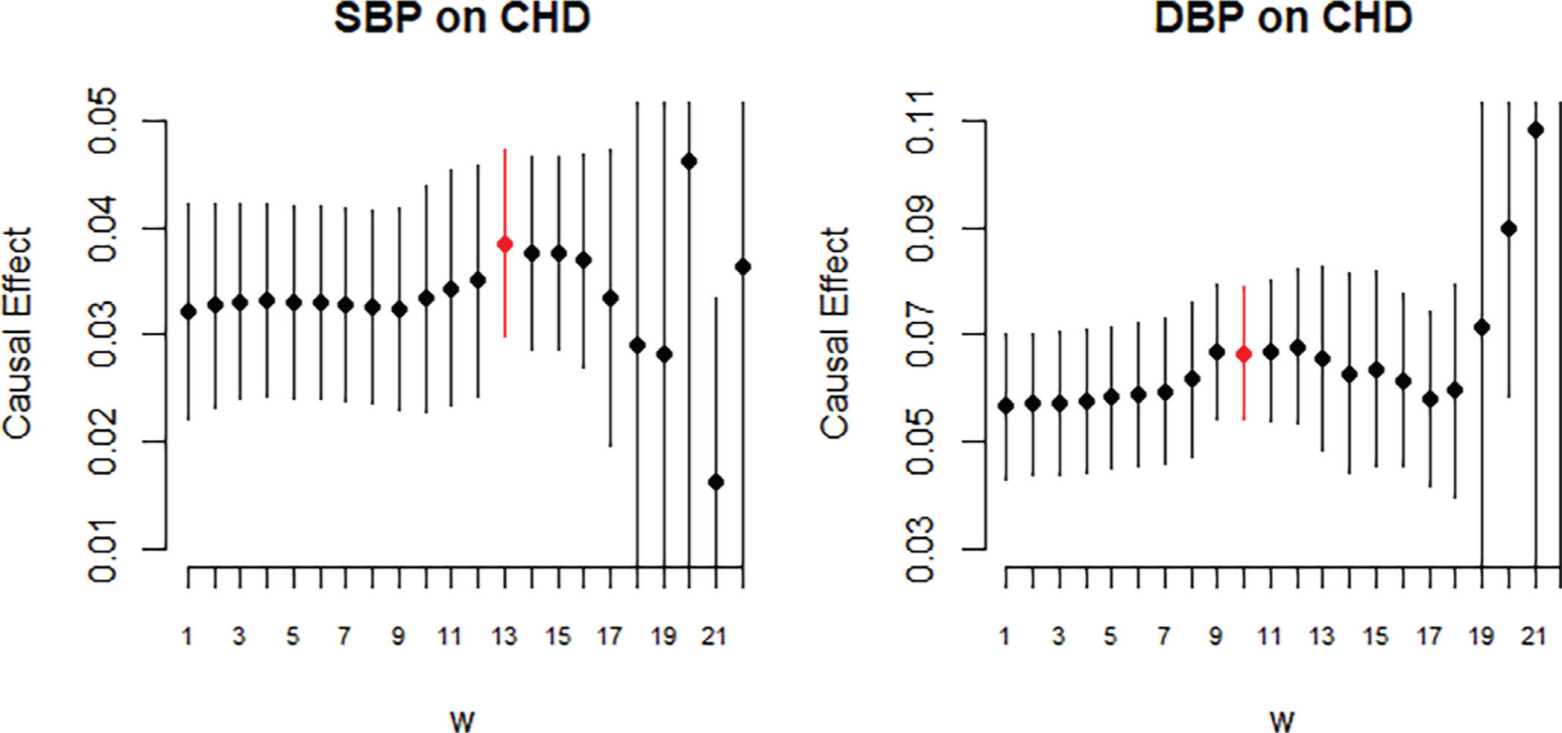
Log-odds ratios of increase in coronary heart disease risk per 1 mmHg increase in systolic and diastolic blood pressure. Point estimates and 95% confidence intervals for JAM-MR implementations with various values of the tuning parameter [Colour figure can be viewed at wileyonlinelibrary.com]

To further explore the effect of pleiotropic variants on the estimated effect of blood pressure on CHD risk, we can use the sensitivity analysis plots of Figure 5. The overall impact of pleiotropy in this application seems rather small, as similar causal effect estimates are obtained for a wide range of *w* values. There is perhaps a slight downward bias for diastolic blood pressure with small *w* values, as we speculated by inspecting the results of Table 3.

As in the simulation study, JAM-MR estimates with unnecessarily large *w* become unstable, with both inaccurate causal effect estimates and increased SEs leading to the behavior observed in the sensitivity plot of Figure 5. The difference between Figures 2 and 5 is discussed in the supplementary material. Briefly, the results reported in Figure 2 were averaged over 1000 replications and the instability in computing the JAM-MR causal effect estimate with large *w* is not directional, therefore it was averaged out across the 1000 iterations and only the inflation in SEs could be observed. Figure 5 was generated based on a single dataset and a single implementation of the algorithm, and is therefore reminiscent of what one would observe by running JAM-MR in real-data applications. The poor performance for certain large values of *w* is evident.

The MR-Egger method rejects the null hypothesis of no causal association and indicates the existence of a causal effect on CHD risk for both blood pressure traits. MR-Egger was the method used to generate the null findings in previous work.^5^ This seeming inconsistency is due to the larger sample sizes and numbers of SNPs that we have used in this article. The previous analysis^5^ was based only on 29 genetic variants that were known to be associated with blood pressure at that point, and its power to detect a causal association was lower.

The mode-based estimation method was implemented using the default function in the R package MendelianRan-domization. The method yielded much larger SEs than other algorithms. This was due to the presence of a few genetic variants with extreme outlying effects in our dataset, which were in turn the result of weak instrument bias. The presence of weak instruments can have adverse effects on the performance of the mode-based approach. In fact, the effects can be more severe than those reported in Table 3. The estimates for diastolic blood pressure in Table 5 were obtained after discarding three obvious outliers from the dataset. Using all 391 genetic variants, the resulting causal effect estimate was −0.063 (95% confidence interval (−22.27,22.152)) for the simple and −0.063 (95% confidence interval (−22.260,22.134)) for the weighted version of the mode-based method; both confidence intervals were too wide to be of practical use. This poor performance of the method was not observed in our simulation study because our simulation design did not generate very weak instruments.

## 5 | DISCUSSION

In this article, we have developed a new algorithm for causal effect estimation in Mendelian randomization when some of the candidate instruments are pleiotropic. Our algorithm uses Bayesian variable selection to identify sets of genetic variants with homogeneous causal effect estimates, and model averaging to obtain causal effect estimates and SEs. A wide range of simulation studies and a real-data application using data from a recent GWAS to study the effect of blood pressure on CHD risk were conducted to evaluate the effectiveness of the JAM-MR algorithm, focusing primarily on implementations in large-scale GWAS studies with hundreds of genetic variants that can explain a decent proportion of variation in the risk factor.

Compared with other approaches for Mendelian randomization with pleiotropic variants, the JAM-MR algorithm offers accurate causal effect estimation but is hindered by a slight reduction in coverage of confidence intervals. This issue makes it difficult to draw causal conclusions using JAM-MR alone, but we believe that the algorithm can still act as a sensitivity analysis tool for Mendelian randomization studies. To that end, the algorithm can facilitate the use of prior information on instrument validity; this point was not discussed extensively in the current paper but we intend to explore it in the future. JAM-MR also yields posterior inclusion probabilities for genetic variants, which can be interpreted as a measure of validity for each variant, and can be used to visualize the impact of pleiotropy in an application, by plotting causal effect estimates and confidence intervals for various *w* values.

JAM-MR also provides a natural framework for incorporating genetic correlations into a Mendelian randomization analysis and selecting the most relevant variants from a densely genotyped region. Common approaches for Mendelian randomization typically assume that genetic variants are independent therefore we have focused on simulations with independent variants in this article. In related work, we are investigating the advantages of utilizing JAM-MR’s variable selection compared with pruning and other approaches for Mendelian randomization with correlated instruments, in order to incorporate multiple correlated effects from regions which harbor complex genetic signals for the trait of interest.

The most notable limitation of our approach is the algorithm’s imperfect calibration of SEs. The inflation of Type I error rates was not as severe as for some of the other Mendelian randomization approaches in many applications: Type I error rates usually ranged between 7% and 10%. If JAM-MR is to be used to assess the existence of a causal relation between a risk factor and an outcome of interest, a heuristic yet practical adjustment could be that researchers report 99% confidence intervals instead of the traditional 95% threshold, when using the algorithm.

It would also be useful to devise an efficient automatic procedure for specifying the tuning parameter *w* that does not have to rely on a grid search. The time required to implement the JAM-MR algorithm is longer than that for other Mendelian randomization approaches: for the simulated datasets with *P* = 400 genetic variants, the running time was about 3 to 5 minutes per value of *w*. A grid search over many values takes longer, but can be implemented more efficiently if researchers have access to cluster computing facilities, as runs for different *w* values can be executed in parallel. Nevertheless, it would be desirable to obtain an accurate causal effect estimate based on a single *w* value.

JAM-MR’s stochastic search procedure is quite flexible and can explore large parameter spaces of causal configurations; as a result, the algorithm is quite efficient when used with large numbers of genetic variants. The stochastic search targets the largest homogeneous set of genetic variants. If smaller sets of genetic variants with homogeneous ratio estimates exist, the stochastic search will sometimes identify them; in practice, this can be checked by inspecting the list of posterior model probabilities. Such sets may correspond to several pleiotropic variants acting on the same causal pathway to the outcome, and the biological interpretation of these sets can be an interesting question in applications. We note however that JAM-MR often requires a very large number of iterations in order to identify such sets. To improve its efficiency at identifying such sets, JAM-MR could benefit from a more elaborate implementation of the reversible-jump procedure, for example, using parallel tempering.

In its current form, the JAM-MR algorithm is not fully Bayesian. Although it utilizes Bayesian variable selection to obtain posterior model probabilities and facilitate model averaging, the process of causal effect estimation for each model is conducted using classical IVW formulas. A fully Bayesian algorithm for Mendelian randomization would require further modeling assumptions for the causal effect parameter and would return a posterior sample for that parameter. This is another interesting potential extension of JAM-MR.

Inconclusion, JAM-MRperformspleiotropy-robustcausaleffectestimationforMendelianrandomization.Inthisarticle, we have introduced the algorithm, evaluated its performance in simulation studies and used it to conduct a Mendelian randomization analysis of the effect of blood pressure on CHD risk, using a recent large-scale blood pressure meta-GWAS. We hope that JAM-MR will become a useful addition to the Mendelian randomization literature.

The JAM-MR algorithm has been implemented in R as part of the GitHub package R2BGLiMS, available at https://github.com/pjnewcombe/R2BGLiMS.

## Supplementary Material

supinfo

## Figures and Tables

**TABLE 1:** Different simulation scenarios

Scenario	Type of pleiotropy	Direct effects	Confounder effects
1	Balanced	*𝛿*_*j*_ ∼ ±*N*(0.7,0.2^2^)	*𝛼*_*j*_ = 0
2	Directional	*𝛿*_*j*_ ∼ *N*(0.7,0.2^2^)	*𝛼*_*j*_ = 0
	InSIDE satisfied		
3	Directional	*𝛿*_*j*_ ∼ *N*(0.7,0.2^2^)	*𝛼*_*j*_ ∼ *N*(0.4,0.2^2^)
	InSIDE violated		

**TABLE 2:** Average causal effect estimates, estimated SEs, root mean squared errors, empirical Type I error rates (*𝜃* = 0), and empirical coverage (*𝜃* = 0.3) for the three simulation scenarios and a variety of MR methods

	Causal effect *𝜽* = 0				Causal effect *𝜽* = 0.3			
		
Method	Mean	SE	RMSE	Type I	Mean	SE	RMSE	Coverage
Balanced pleiotropy								
IVW	0.000	0.022	0.022	0.057	0.296	0.022	0.023	0.942
MR-Egger	−0.003	0.065	0.065	0.055	0.265	0.066	0.077	0.912
Median	0.000	0.010	0.010	0.061	0.294	0.012	0.014	0.915
Mode	0.000	0.016	0.012	0.011	0.288	0.020	0.019	0.954
Lasso	0.000	0.007	0.007	0.089	0.296	0.008	0.010	0.861
MR-Presso	0.000	0.007	0.008	0.086	0.296	0.010	0.012	0.884
MR-Raps	0.000	0.019	0.019	0.048	0.300	0.020	0.020	0.951
ConMix	0.000	–	0.008	0.218	0.298	–	0.011	0.778
JAM-MR	0.000	0.007	0.008	0.074	0.294	0.009	0.013	0.851
Oracle	0.000	0.007	0.007	0.045	0.296	0.008	0.009	0.919
Directional pleiotropy, InSIDE satisfied								
IVW	0.208	0.019	0.208	1.000	0.503	0.019	0.204	0.000
MR-Egger	0.000	0.056	0.058	0.067	0.264	0.057	0.068	0.910
Median	0.052	0.011	0.053	0.999	0.358	0.013	0.059	0.008
Mode	0.000	0.014	0.011	0.012	0.290	0.018	0.017	0.961
Lasso	0.028	0.007	0.029	0.948	0.339	0.008	0.041	0.021
MR-Presso	0.077	0.011	0.078	1.000	0.396	0.013	0.098	0.000
MR-Raps	0.170	0.019	0.171	1.000	0.472	0.020	0.173	0.000
ConMix	0.001	–	0.008	0.183	0.302	–	0.011	0.771
JAM-MR	0.005	0.007	0.009	0.106	0.301	0.009	0.012	0.897
Oracle	0.000	0.007	0.006	0.044	0.296	0.008	0.009	0.919
Directional pleiot	ropy, InSIDE	violated						
IVW	0.372	0.022	0.372	1.000	0.668	0.023	0.369	0.000
MR-Egger	0.705	0.060	0.707	1.000	0.978	0.062	0.680	0.000
Median	0.167	0.018	0.175	1.000	0.496	0.021	0.204	0.000
Mode	0.000	0.017	0.011	0.016	0.288	0.016	0.018	0.929
Lasso	0.045	0.008	0.047	0.996	0.366	0.009	0.068	0.000
MR-Presso	0.204	0.017	0.206	1.000	0.517	0.018	0.218	0.000
MR-Raps	0.338	0.024	0.339	1.000	0.640	0.024	0.340	0.000
ConMix	0.001	–	0.009	0.220	0.299	–	0.010	0.813
JAM-MR	0.002	0.007	0.008	0.074	0.296	0.009	0.011	0.899
Oracle	0.000	0.007	0.007	0.053	0.296	0.008	0.009	0.919

**TABLE 3:** Log-odds ratios of increase in coronary heart disease risk per 1 mmHg increase in the corresponding blood pressure measurement

	Systolic Blood Pressure		Diastolic Blood Pressure	
		
Method	Estimate	95% CI	Estimate	95% CI
IVW	0.034	(0.026, 0.041)	0.048	(0.035, 0.061)
MR-Egger	0.024	(0.008, 0.041)	0.083	(0.058, 0.109)
Median S	0.038	(0.030, 0.046)	0.053	(0.038, 0.068)
Median W	0.032	(0.024, 0.040)	0.060	(0.047, 0.073)
Mode S	0.026	(−0.087, 0.139)	0.061	(−0.059, 0.181)
Mode W	0.026	(−0.087, 0.139)	0.061	(−0.057, 0.179)
Lasso	0.037	(0.032, 0.042)	0.048	(0.040, 0.057)
MR-Presso	0.038	(0.032, 0.045)	0.052	(0.042, 0.063)
MR-Raps	0.039	(0.032, 0.045)	0.055	(0.043, 0.066)
ConMix	0.038	(0.031, 0.043)	0.051	(0.040, 0.063)
JAM-MR	0.039	(0.030, 0.047)	0.067	(0.054, 0.079)

*Note:* Causal effect estimates and 95% confidence intervals for a variety of MR methods.
