# Supplementary material for: Bayesian variable selection with a pleiotropic loss function in Mendelian randomization
Source: Stat Med. Author manuscript; Available in PMC 2022 Oct 15. (PMC8446304; doi:10.1002/sim.9109)
Supplement: supinfo [file NIHMS1714125-supplement-supinfo.pdf]

Bayesian variable selection with a pleiotropic loss function in Mendelian randomization

Apostolos Gkatzionis*[1,2], Stephen Burgess[1,3], David V Conti[4], Paul J Newcombe[1]

[1]MRC Biostatistics Unit, University of Cambridge, Cambridge, United Kingdom. [2]MRC Integrative Epidemiology Unit, University of Bristol, Bristol, United Kingdom. [2]Department of Public Health and Primary Care, School of Clinical Medicine, University of Cambridge, Cambridge, United Kingdom. [3]Department of Preventive Medicine, Keck School of Medicine, University of Southern California, California, USA. *Corresponding author. Email: apostolos.gkatzionis@bristol.ac.uk.

Supplementary Material

SIMULATION DESIGN

Figure 1 illustrates the design of our simulation study. We plot a histogram of the negative logarithms of univariate p-values generated for each genetic variant (left) and a histogram of univariate causal effect estimates obtained from all the simulated genetic variants (right). The two histograms are constructed using data from all 1000 replications of simulation scenario 2 with $\theta = 0$. Similar plots were obtained for the other two simulation scenarios, as well as for $\theta = 0.3$.

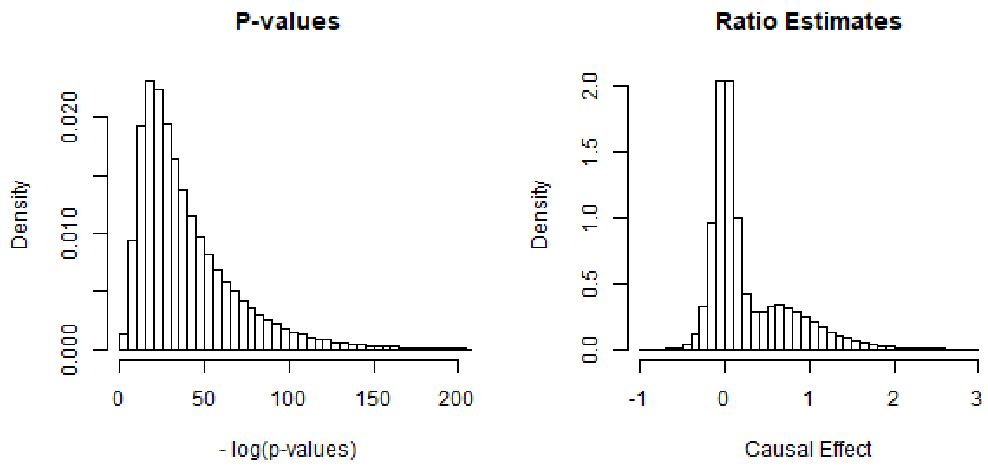

FIGURE 1 Histogram of univariate p-values of association between each genetic variant and the risk factor (left) and univariate causal effect estimates (right) over all replications of simulation scenario 2 with $\theta = 0$.

COMPETING METHODS

Mendelian randomization in the presence of invalid instruments is a very active area of research. A wide range of statistical techniques have been used to either identify the pleiotropic variants and remove them from the analysis, or robustify the process of causal effect estimation. Here we give a brief description of the methods used in our simulations.

One of the earliest and most widely used approaches is MR-Egger regression[1]. Like the inverse-variance weighted estimate, MR-Egger regression is motivated from the meta-analysis literature. Loosely, it consists of fitting a linear regression of the SNP-outcome association estimates on the SNP-exposure estimates: $\hat{\beta}_{Yj} = \alpha + \hat{\beta}_{Xj}\theta + \epsilon$. The IVW estimate is obtained when $\alpha = 0$. When $\alpha \neq 0$, the intercept models the aggregate pleiotropic contribution of all SNPs on the outcome. MR-Egger yields consistent causal effect estimates under the assumption that instrument strength is independent of the genetic variants' direct effects on the outcome (InSIDE). However, as illustrated in our simulation study, the method's power to detect causal associations is often quite low.

Another common approach is to estimate the causal effect of interest by the (weighted or unweighted) median of univariate estimates for all available genetic variants. This median estimator is robust to outlying pleiotropic effects and is asymptotically unbiased if more than 50% of the available genetic variants are valid instruments.

Mode-based estimation[2] fits a kernel density to the univariate causal effect estimates and uses the mode of that density as the overall estimate; standard errors are then computed based on a bootstrap procedure. This approach weakens the majority assumption of the median and yields accurate causal effect estimates under the assumption that only a plurality of genetic instruments are valid.

Lasso regularization has also been considered in order to identify which genetic variants exhibit pleiotropic effects. This was first proposed in the presence of individual-level data[3] and later adapted to situations where only summary-level data are available[4]. The latter paper[4] also developed robust regression techniques for causal effect estimation.

Another approach that relies on variable selection is MR-Presso[5], which performs outlier detection and deletion by conducting a hypothesis test of pleiotropy for each genetic variant.

The contamination mixture approach[6] uses a mixture model for the univariate causal effect estimates. The mixture contains two components: valid instruments are assumed to be normally distributed around the true causal effect and invalid instruments are normally distributed around zero. The method constructs a likelihood based on that mixture model, and the likelihood is maximized over both parameter values and assignments of SNPs to the two mixture components; the maximization relies on profile likelihood techniques.

The MR-Raps method[7] assumes a random-effects distribution for pleiotropic effects, and also relies on profile likelihood and robust regression techniques for causal effect estimation.

For an extensive comparison of pleiotropy-robust Mendelian randomization algorithms using summary data, we refer the reader to two recent review papers[8,9].

The list of methods that we considered in our simulation study is not exhaustive. For example, we did not implement the heterogeneity penalization approach[10]. Similar to JAM-MR, this approach relies on model averaging, but instead of our algorithm's stochastic search it uses an exhaustive search over possible models, and is therefore only applicable for small numbers of genetic variants. We have also not considered the mixture modelling method MRMix[11]; this method was shown to perform well in terms of Type I error calibration in a recent study[9], but in order to function efficiently it also requires access to summary statistics for additional genetic variants throughout the genome that are not associated with the risk factor. We did not generate such variants in our simulations, since it would have dramatically increased their computational cost. Finally, we did not utilize methods requiring the availability of individual-level data, such as sisVIVE[3] or an instrumental variables method[12] which also uses Reversible-Jump MCMC.

IMPLEMENTATION OF THE VARIOUS METHODS IN THE SIMULATION STUDY

For the implementation of the various Mendelian randomization methods in our simulation study, we used available `R` packages. In particular, we used the packages `MendelianRandomization` (for the IVW, MR-Egger, contamination mixture, median and mode-based methods), `MRPRESSO` (for MR-Presso) and `mr.raps` (for MR-Raps). For the Lasso method, we used the `R` code provided in the Appendix of the relevant paper[4].

For the median and mode-based methods we implemented the weighted version. Results using the unweighted versions are not reported here but were quite similar to those for the weighted implementations in all our simulation scenarios. For the lasso method we used the "heterogeneity" approach[4] to specify a value for the tuning parameter. MR-Raps was implemented using the Tukey loss function and an overdispersed model. For the other methods, we used the default settings in the corresponding R packages.

For the implementation of JAM-MR, we used a grid search consisting of 50 points, evenly scaled on a logarithmic scale from $w = 0.01N_1$ to $w = 100N_1$, plus an execution for $w = 0$ (the original JAM algorithm with no pleiotropy penalization). In each instance, the algorithm was run for 1 million iterations. Since genetic variants were assumed to be independent, we did not require a reference dataset for the matrix of genetic correlations. Instead, the matrix $G^T G$ was set as follows. The off-diagonal elements were set equal to zero, reflecting our assumption of independent genetic variants. The diagonal elements were computed by $(G^T G)_{jj} = 2N_1 \hat{f}_j(1 - \hat{f}_j)$, where $\hat{f}_j$ is the estimated effect allele frequency for variant G_j. Finally, JAM-MR's model-specific estimates were computed using the truncated multiplicative random effects approach.

MOTIVATION FOR THE TRUNCATED NORMAL RANDOM-EFFECTS MODEL

Here we provide a simulation to illustrate why the use of IVW estimates to compute model-specific causal effects and standard errors may be suboptimal, and motivate the use of the truncated multiplicative random-effects model. Consider the simulation plotted in Figure 2. The figure was created using a simulation model similar to the directional pleiotropy scenario with no violations of the InSIDE assumption in our main simulations. It plots univariate causal effect estimates and standard errors for $P = 50$ genetic variants. Variants 1-35 were generated as valid instruments and variants 36-50 were generated to be pleiotropic. The true causal effect of the risk factor on the outcome was set to zero. JAM-MR was implemented to identify variants suitable for inclusion in a Mendelian randomization analysis. The variants that were assigned a posterior inclusion probability lower than 0.5 are coloured red.

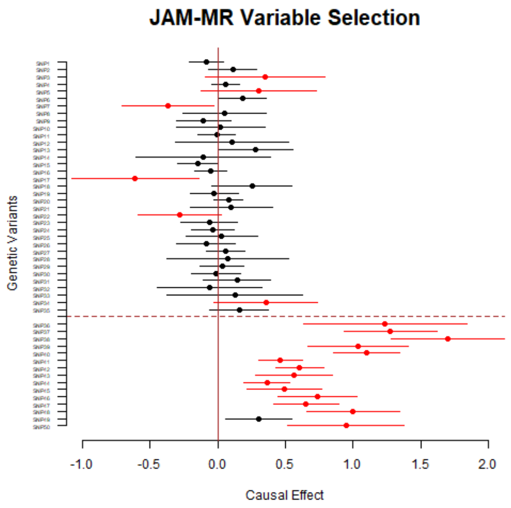

FIGURE 2 JAM-MR variable selection in a simulated dataset of 50 genetic variants. A single implementation of a directional pleiotropy simulation (scenario 2) with $\theta = 0$. Causal effect estimates and 95% confidence intervals for each variant are plotted. Variants above the dotted line were simulated as valid and variants below the dotted line were simulated as pleiotropic. Variants assigned a posterior inclusion probability lower than 50% are coloured red.

The plot contains some variants that were simulated to be valid instruments, but whose univariate causal effect estimates were located far from the true value of $\theta = 0$ due to random variation. Since JAM-MR uses heterogeneity as a proxy for pleiotropic behaviour, it will interpret such variants as pleiotropic and assign them a relatively small posterior inclusion probability. On

the other hand, variants simulated to be invalid but with only moderate pleiotropic effects may be assigned a high inclusion probability due to the proximity of their causal effect estimates to those of valid variants.

The truncated normal multiplicative random effects model is designed to account for this difference between JAM-MR's heterogeneity penalization and the normality assumption underlying the IVW estimates. The use of truncation models the downweighting of valid variants with outlying effects and the use of a random-effects model accounts for the upweighting of moderately pleiotropic variants.

COMPARISON BETWEEN APPROACHES FOR COMPUTING JAM-MR MODEL-SPECIFIC ESTIMATES

Here we compare three approaches to computing model-specific estimates and standard errors when averaging over the models visited by JAM-MR to compute an overall causal effect estimate. The three approaches to be compared are (a) the standard inverse-variance weighted estimate, (b) the IVW estimate using a multiplicative random-effects assumption to compute the standard error, and (c) the truncated normal multiplicative random-effects model described in Section 2.2.5 of the main paper.

Each of the three approaches was implemented in the simulation scenarios of Section 3 of the paper and causal effect estimates and standard errors were obtained. The results are summarized in Table 1 for all three simulation scenarios. We report average causal effect estimates, estimated standard errors, root mean squared errors and Type I error rates.

TABLE 1 Comparison between the fixed-effects ("IVW"), multiplicative random-effects ("Mult") and truncated multiplicative random-effects ("Trunc") approaches for computing model-specific JAM-MR causal effect estimates.

Method	$\theta = 0$				$\theta = 0.3$			
	Mean	SE	RMSE	Type I	Mean	SE	RMSE	Type I
Balanced Pleiotropy								
IVW	0.000	0.006	0.022	0.633	0.296	0.007	0.023	0.581
Mult	0.000	0.007	0.009	0.134	0.294	0.008	0.013	0.256
Trunc	0.000	0.007	0.008	0.074	0.294	0.009	0.013	0.149
Directional Pleiotropy, InSIDE Satisfied								
IVW	0.206	0.006	0.207	1.000	0.502	0.007	0.202	1.000
Mult	0.007	0.007	0.011	0.240	0.305	0.008	0.012	0.208
Trunc	0.005	0.007	0.009	0.106	0.301	0.009	0.012	0.103
Directional Pleiotropy, InSIDE Violated								
IVW	0.372	0.005	0.372	1.000	0.669	0.006	0.369	1.000
Mult	0.003	0.007	0.009	0.126	0.298	0.008	0.011	0.137
Trunc	0.002	0.007	0.008	0.074	0.296	0.009	0.011	0.101

The table illustrates the benefit provided by using the truncated approach, as it offers unbiased causal effect estimation and reasonable Type I error rates. The multiplicative random-effects approach is also able to accurately estimate the causal effect but has inflated Type I error rates in comparison. On the other hand, using the default IVW approach yields poor results. This is because the IVW approach does not combine well with JAM-MR's minimum-standard-error tuning process. When implementing JAM-MR with the standard IVW model-specific estimates, the run with $w = 0$ is often selected as the one with the smallest causal standard error. For $w = 0$, the algorithm implements no pleiotropy penalization and biased estimates are obtained. If we fix w instead of implementing a grid search, the fixed-effects IVW method performs similar to the multiplicative random-effects approach - in fact, the two methods give the same causal effect estimate[13]. In any case, truncation is the most promising approach.

BEHAVIOUR OF JAM-MR FOR LARGE *W*

We now discuss the performance of JAM-MR when using a large value for the tuning parameter w. First, we show that the model indicator γ minimizing the loss function $\ell_1(\hat{\theta}, \gamma) = \frac{1}{P_\gamma - 1} \sum_{j \in R_\gamma} \left(\hat{\theta}_j - \hat{\theta}_{\gamma,IVW}\right)^2$ is the two-SNP model $\gamma = \{\hat{\theta}_{j_1}, \hat{\theta}_{j_2}\}$ where j_1, j_2 are the two variants for which $\left|\hat{\theta}_{j_1} - \hat{\theta}_{j_2}\right|$ is smallest.

To start, observe that the loss function $\ell_1(\hat{\theta}, \gamma)$ can be interpreted as the sample variance of the observations $\hat{\theta}_j \, : \, j \in \boldsymbol{R}_\gamma$ using a weighted mean of these observations (the IVW estimate) instead of the sample mean. The weighted loss function $\ell_2(\hat{\theta}, \gamma)$ can be given a similar interpretation as a weighted sample variance. Note that by the term "sample variance" we do not refer to the squared standard error of the IVW estimator, which is given by Equation (18) in the main part of our paper.

We then note that for any three numbers x_1, x_2, x_3,

$$Var\left(\{x_1, x_2, x_3\}\right) \leq \min\left\{Var\left(\{x_1, x_2\}\right), Var\left(\{x_1, x_3\}\right), Var\left(\{x_2, x_3\}\right)\right\}$$

as can be shown by writing down the expression for the sample variance and some algebraic calculations. The same can be shown for more than three observations, by mathematical induction:

$$Var\left(\{x_1, x_2, \dots, x_n\}\right) \leq \min_{i,j=1,\dots,n, i \neq j} Var\left(\{x_i, x_j\}\right)$$

Similar arguments can be used to show that the same applies for the weighted sample variances ℓ_1 and ℓ_2. In practice this means that for any model γ, we can find a sub-model consisting of only two genetic variants for which the value of the two loss functions is smaller. The model that minimizes the loss function is the model containing only the two SNPs j_1, j_2 for which the difference in univariate causal effect estimates $|\hat{\theta}_{j_1} - \hat{\theta}_{j_1}|$ is the smallest. In addition, note that w determines the relative impact of the loss function on JAM-MR's variable selection relative to the JAM likelihood and the prior. Asymptotically as $w \rightarrow \infty$, the variable selection is dictated by the loss function, which means that the algorithm will prioritize that model. For large but finite values of w, the algorithm's stochastic nature means that it will concentrate on small models containing SNPs with very similar univariate ratio estimates.

This explains the performance of JAM-MR's variable selection for large w. If two SNPs (or a small number of SNPs) happen to yield very similar causal effect estimates, these SNPs will be artificially upweighted by the algorithm. This can happen by chance, meaning that the two similar SNPs may be pleiotropic, or (more likely) they may be valid but their causal effect estimates may be either slightly larger or slightly smaller than the true causal effect value due to random variation. This added variability in causal effect estimates is mostly not directional (there is a limited directionality towards the pleiotropic direction) and was hence averaged out across many replications in our simulation study. this can be confirmed by inspecting Figure 2 of the paper, where average causal effect estimates for large w values were close to the true value, but there was increased uncertainty around each estimate. On the other hand, when a single implementation of JAM-MR is executed, runs of the algorithm with large w values may yield inaccurate causal effect estimates as shown in Figure 5 of the paper, where both the causal effect estimates and the standard errors are affected as w grows.

ADDITIONAL SIMULATIONS

Summary of additional simulations implemented

In this section we report simulation results from additional simulations, not included in the main part of the paper. To assess the robustness of JAM-MR and other Mendelian randomization algorithms to a variety of simulation scenarios, we performed four additional sets of simulations. First, we modified the sample sizes of the two GWAS studies, the number of genetic variants included in the Mendelian randomization analysis and the proportion of variation in the risk factor explained by the genetic variants. Specifically, we considered three settings:

- $N_1 = N_2 = 50000$ individuals in each GWAS and $P = 50$ genetic variants that explain 3% of the genetic variation in the risk factor.
- $N_1 = N_2 = 100000$ individuals in each GWAS and $P = 100$ genetic variants that explain 5% of the genetic variation in the risk factor.

- $N_1 = N_2 = 200000$ individuals in each GWAS and $P = 200$ genetic variants that explain 7% of the genetic variation in the risk factor.

In the second set of simulations, we modified the proportion of genetic variants with pleiotropic effects. We used the default simulation settings ($N_1 = N_2 = 300000$, $P = 400$ and 10% variation in the risk factor explained) and generated three sets of simulations, with 0%, 20% and 50% of the genetic variants being invalid respectively.

In the third set of simulations, we considered a pleiotropic scenario with less strong directional pleiotropy than in the main body of the paper: specifically, we simulated 80 of the 120 pleiotropic variants to have risk-increasing effects on the outcome and the remaining 40 variants to have risk-decreasing effects. This is a compromise between our "balanced pleiotropy" scenario (where 60 genetic variants had risk-increasing and 60 had risk-decreasing effects) and our original "directional pleiotropy" scenario (where all 120 pleiotropic variants were generated to have risk-increasing effects) and resembles the real-data application presented in our manuscript.

Finally, we assessed the performance of JAM-MR and other Mendelian randomization approaches in simulations in which the risk factor and outcome values were not generated from normal distributions. Specifically, we assessed robustness to non-normality of the confounder U, risk factor X and outcome Y by modifying the distribution of the confounder residual, the risk factor residual and the outcome residual respectively.

To reduce the computational burden, we only implemented 500 replications of each simulation. For JAM-MR, we also reduced the grid size to 26 points (compared to 51 points for simulations in the main part of the paper). The results of these simulations are reported in Tables 2-15.

Different GWAS sample sizes and numbers of genetic variants

The simulations of Tables 2-4 illustrate that the performance of JAM-MR deteriorates when the GWAS sample sizes and proportion of variation in the risk factor are small. The algorithm performed rather poorly in the directional pleiotropy simulations of Table 2, where $N_1 = N_2 = 50000$ and only 3% variation in the risk factor was explained by the SNPs included in the analysis. Both the bias and the coverage properties of confidence intervals were affected. Some of the competing methods were subject to larger biases still, and the mode-based method was a clear winner in this scenario.

In the simulations of Table 3 ($N_1 = N_2 = 100000$ and 5% variation in the risk factor explained by the SNPs), the performance of JAM-MR improved and was close to that observed in the simulations in the main part of the paper, with slightly worse coverage properties in some settings. Further improvement was observed in the simulations of Table 4 ($N_1 = N_2 = 200000$ and 7% variation in the risk factor explained by the SNPs). These results seem to suggest that JAM-MR is more suitable for Mendelian randomization analyses of complex polygenic traits, for which large meta-GWAS have been conducted on hundreds of thousands of individuals and hundreds of associated genetic variants have been identified.

We note that Type I error rates for some of the competing methods were better in simulations with smaller sample sizes than in the original simulation with $N_1 = N_2 = 300000$ individuals. Using larger sample sizes in our simulation study gave rise to quite narrow confidence intervals, and the bias exhibited by these methods implied that the confidence intervals were centered at the wrong estimates, therefore Type I error rates were inflated. Although it is perhaps counterintuitive that some methods may perform better with smaller sample sizes, similar observations have been made elsewhere[9].

Different proportions of pleiotropic SNPs

The results of simulations with varying numbers of pleiotropic instruments are reported in Tables 5-7. First, we report results from the simulation with no invaild instruments, for comparison. These results are reported in Table 5. As would be expected, we observed minimal bias for most MR methods, especially for a null causal effect. In this simulation, the IVW and oracle methods coincide, while the median, Lasso, MR-Presso, MR-Raps and JAM-MR methods yielded similar mean estimates and standard errors as IVW. The mode-based method and MR-Egger were more conservative, having larger standard errors (and a small downward bias in the case of MR-Egger with a nun-null causal effect). Coverage figures for most methods ranged from nominal to slightly reduced; JAM-MR had near-nominal coverage under a null effect and slightly reduced for $\theta = 0.3$. The contamination mixture's relatively poor performance can be explained by the fact that the default parameter values used by the method were not suitable for the datasets we generated. Overall, the performance of most methods was slightly worse under a non-null causal effect. This could perhaps be explained by weak instrument bias, which acts towards the null in two-sample MR analyses, as discussed in the main paper.

TABLE 2 Simulation results with sample sizes of $N_1 = N_2 = 50000$ and $P = 50$ genetic variants that explain 3% genetic variation in the risk factor.

Method	Causal Effect $\theta = 0$				Causal Effect $\theta = 0.3$			
	Mean	SE	RMSE	Type I	Mean	SE	RMSE	Coverage
Balanced Pleiotropy								
IVW	0.005	0.064	0.064	0.054	0.287	0.066	0.071	0.916
MR-Egger	-0.010	0.181	0.185	0.068	0.220	0.188	0.214	0.920
Median	0.001	0.044	0.046	0.060	0.281	0.052	0.059	0.912
Mode	0.000	0.066	0.047	0.016	0.264	0.078	0.070	0.958
Lasso	0.002	0.029	0.044	0.192	0.288	0.033	0.058	0.742
MR-Presso	0.002	0.036	0.046	0.124	0.288	0.044	0.061	0.830
MR-Raps	0.005	0.061	0.060	0.060	0.297	0.065	0.066	0.936
ConMix	0.001	—	0.042	0.078	0.299	—	0.061	0.888
JAM-MR	0.000	0.046	0.057	0.094	0.278	0.054	0.069	0.870
Oracle	0.001	0.031	0.029	0.040	0.289	0.038	0.037	0.958
Directional Pleiotropy, InSIDE Satisfied								
IVW	0.205	0.057	0.208	1.000	0.497	0.061	0.202	0.030
MR-Egger	0.004	0.161	0.169	0.066	0.220	0.168	0.194	0.916
Median	0.085	0.046	0.099	0.430	0.387	0.055	0.104	0.674
Mode	0.011	0.066	0.044	0.016	0.293	0.075	0.058	0.968
Lasso	0.074	0.029	0.086	0.654	0.389	0.034	0.103	0.344
MR-Presso	0.116	0.041	0.124	0.804	0.434	0.049	0.143	0.210
MR-Raps	0.177	0.059	0.181	0.958	0.485	0.063	0.190	0.082
ConMix	0.010	—	0.045	0.102	0.351	—	0.131	0.774
JAM-MR	0.064	0.069	0.120	0.270	0.368	0.062	0.110	0.684
Oracle	-0.001	0.031	0.030	0.044	0.291	0.038	0.038	0.954
Directional Pleiotropy, InSIDE Violated								
IVW	0.365	0.065	0.368	1.000	0.656	0.068	0.360	0.000
MR-Egger	0.629	0.175	0.654	0.924	0.876	0.185	0.605	0.150
Median	0.262	0.058	0.295	0.932	0.570	0.066	0.299	0.114
Mode	0.012	0.066	0.058	0.018	0.297	0.096	0.081	0.968
Lasso	0.115	0.032	0.128	0.844	0.444	0.037	0.164	0.164
MR-Presso	0.229	0.051	0.239	0.988	0.546	0.055	0.255	0.016
MR-Raps	0.341	0.070	0.345	1.000	0.641	0.073	0.345	0.000
ConMix	0.012	—	0.097	0.072	0.409	—	0.291	0.822
JAM-MR	0.261	0.062	0.380	0.536	0.575	0.067	0.383	0.358
Oracle	0.001	0.031	0.029	0.024	0.291	0.038	0.036	0.952

Table 6 contains the results of simulations with 20% invalid instruments. The performance of all methods was slightly worse than that in Table 5 and slightly better than that reported in the main part of our paper, where 30% of the genetic instruments were invalid. The performance of JAM-MR was in line with this trend: our algorithm was again one of the most accurate approaches for causal effect estimation, at the cost of a small amount of undercoverage for confidence intervals. The latter was more pronounced for $\theta = 0.3$.

The simulation of Table 7 (50% invalid instruments) was quite challenging. JAM-MR was still able to offer accurate causal effect estimates in two out of the three scenarios, but failed to do so in the scenario where the InSIDE assumption was violated. We note that JAM-MR does not depend on the InSIDE assumption. The reason for its failure was that the SNP-confounder effects in our simulations were generated to be positive ($\delta_j \sim N(0.4, 0.2^2)$). As a result, in the last simulation of Table 7, the pleiotropic variants were equal in number to the valid SNPs and were stronger instruments on average. In addition, univariate causal effect estimates for the pleiotropic variants were fairly homogeneous in that simulation. This led JAM-MR to identify the

TABLE 3 Simulation results with sample sizes of $N_1 = N_2 = 100000$ and $P = 100$ genetic variants that explain 5% genetic variation in the risk factor.

Method	Causal Effect $\theta = 0$				Causal Effect $\theta = 0.3$			
	Mean	SE	RMSE	Type I	Mean	SE	RMSE	Coverage
Balanced Pleiotropy								
IVW	-0.002	0.044	0.047	0.064	0.296	0.045	0.046	0.928
MR-Egger	-0.005	0.130	0.137	0.068	0.253	0.132	0.142	0.918
Median	-0.001	0.025	0.027	0.076	0.293	0.030	0.032	0.942
Mode	0.000	0.032	0.026	0.018	0.283	0.042	0.037	0.964
Lasso	-0.001	0.016	0.022	0.146	0.294	0.019	0.026	0.850
MR-Presso	-0.002	0.018	0.022	0.120	0.296	0.024	0.030	0.878
MR-Raps	-0.002	0.041	0.042	0.052	0.302	0.043	0.041	0.954
ConMix	-0.001	—	0.021	0.114	0.300	—	0.027	0.878
JAM-MR	-0.001	0.021	0.024	0.092	0.291	0.025	0.033	0.874
Oracle	-0.001	0.017	0.017	0.048	0.295	0.020	0.020	0.952
Directional Pleiotropy, InSIDE Satisfied								
IVW	0.207	0.039	0.208	1.000	0.502	0.040	0.204	0.000
MR-Egger	0.005	0.112	0.114	0.052	0.253	0.116	0.131	0.918
Median	0.067	0.026	0.072	0.708	0.372	0.032	0.080	0.382
Mode	0.004	0.033	0.025	0.014	0.291	0.040	0.032	0.974
Lasso	0.044	0.017	0.050	0.678	0.358	0.020	0.066	0.250
MR-Presso	0.090	0.024	0.094	0.956	0.412	0.029	0.117	0.046
MR-Raps	0.173	0.039	0.175	1.000	0.478	0.041	0.180	0.000
ConMix	0.004	—	0.022	0.122	0.310	—	0.031	0.858
JAM-MR	0.015	0.021	0.034	0.166	0.320	0.027	0.051	0.808
Oracle	0.000	0.017	0.017	0.042	0.295	0.020	0.021	0.930
Directional Pleiotropy, InSIDE Violated								
IVW	0.372	0.046	0.374	1.000	0.666	0.046	0.367	0.000
MR-Egger	0.687	0.124	0.699	1.000	0.946	0.127	0.660	0.002
Median	0.215	0.038	0.240	1.000	0.541	0.044	0.261	0.006
Mode	0.001	0.028	0.022	0.004	0.287	0.037	0.030	0.972
Lasso	0.068	0.019	0.076	0.840	0.397	0.022	0.106	0.074
MR-Presso	0.206	0.034	0.213	1.000	0.526	0.036	0.231	0.000
MR-Raps	0.342	0.049	0.344	1.000	0.642	0.049	0.344	0.000
ConMix	0.001	—	0.020	0.074	0.302	—	0.027	0.904
JAM-MR	0.027	0.022	0.123	0.096	0.358	0.028	0.189	0.822
Oracle	0.000	0.017	0.017	0.036	0.295	0.020	0.021	0.950

set of pleiotropic SNPs as “valid" and downweight the valid genetic variants instead. This is a difficult setting for any Mendelian randomization method. Indeed, the mode was the only approach that managed to identify the true causal effect in all scenarios.

In additional simulations, not reported here, we obtained better performance for JAM-MR when the SNP-confounder effets were simulated to be negative ($\delta_j \sim N(0.4, 0.2^2)$). On the other hand, the mode-based method seemed to struggle in that scenario because the SNP-risk factor and SNP-confounder effects acted on opposite directions and this effectively made the genetic instruments weaker, causing similar problems to the method as those observed in the real-data application.

TABLE 4 Simulation results with sample sizes of $N_1 = N_2 = 200000$ and $P = 200$ genetic variants that explain 7% genetic variation in the risk factor.

Method	Causal Effect $\theta = 0$				Causal Effect $\theta = 0.3$			
	Mean	SE	RMSE	Type I	Mean	SE	RMSE	Coverage
**Balanced Pleiotropy**								
IVW	0.002	0.031	0.032	0.062	0.298	0.031	0.032	0.926
MR-Egger	0.007	0.092	0.093	0.062	0.261	0.093	0.104	0.916
Median	0.001	0.015	0.015	0.060	0.293	0.018	0.020	0.922
Mode	0.001	0.023	0.016	0.008	0.287	0.026	0.023	0.970
Lasso	0.001	0.010	0.010	0.064	0.296	0.011	0.015	0.872
MR-Presso	0.001	0.011	0.012	0.064	0.297	0.014	0.018	0.866
MR-Raps	0.002	0.028	0.027	0.040	0.301	0.029	0.028	0.954
ConMix	0.000	—	0.011	0.112	0.298	—	0.016	0.836
JAM-MR	0.000	0.011	0.012	0.066	0.294	0.014	0.018	0.858
Oracle	0.000	0.010	0.009	0.032	0.296	0.012	0.013	0.922
**Directional Pleiotropy, InSIDE Satisfied**								
IVW	0.208	0.027	0.209	1.000	0.503	0.028	0.204	0.000
MR-Egger	0.003	0.079	0.083	0.058	0.261	0.080	0.093	0.912
Median	0.054	0.016	0.057	0.944	0.361	0.020	0.064	0.104
Mode	0.000	0.018	0.015	0.014	0.289	0.024	0.021	0.966
Lasso	0.029	0.010	0.032	0.742	0.342	0.012	0.046	0.142
MR-Presso	0.082	0.016	0.084	1.000	0.404	0.019	0.106	0.000
MR-Raps	0.171	0.027	0.172	1.000	0.474	0.028	0.175	0.000
ConMix	0.001	—	0.012	0.146	0.303	—	0.016	0.840
JAM-MR	0.005	0.011	0.013	0.088	0.303	0.014	0.022	0.888
Oracle	-0.001	0.010	0.010	0.032	0.296	0.012	0.012	0.942
**Directional Pleiotropy, InSIDE Violated**								
IVW	0.372	0.032	0.373	1.000	0.666	0.032	0.367	0.000
MR-Egger	0.705	0.086	0.710	1.000	0.974	0.088	0.679	0.000
Median	0.182	0.026	0.198	1.000	0.509	0.030	0.224	0.000
Mode	0.000	0.017	0.013	0.008	0.287	0.022	0.022	0.954
Lasso	0.050	0.011	0.053	0.950	0.370	0.013	0.074	0.020
MR-Presso	0.207	0.024	0.211	1.000	0.522	0.025	0.225	0.000
MR-Raps	0.340	0.034	0.341	1.000	0.639	0.034	0.340	0.000
ConMix	0.001	—	0.011	0.124	0.300	—	0.016	0.866
JAM-MR	0.002	0.011	0.011	0.046	0.297	0.014	0.036	0.918
Oracle	0.000	0.010	0.009	0.024	0.295	0.012	0.013	0.928

JAM-MR with small sample sizes

The performance of JAM-MR with smaller sample sizes and fewer genetic instruments (Table 2 was not very accurate. To explore the performance of the method further, we conducted an additional simulation. We used scenario 2 (directional pleiotropy, InSIDE satisfied) and generated three datasets with the following specifications:

- $N_1 = N_2 = 50000$ individuals in each GWAS and $P = 50$ genetic variants that explain 3% of the genetic variation in the risk factor. This is identical to scenario 2 of Table 2 hence the results reported are the same. It resembles a standard Mendelian randomization analysis for a complex trait, using GWAS studies with relatively small sample sizes.

TABLE 5 Simulation results with the default settings and all the genetic instruments being valid.

Method	Causal Effect $\theta = 0$				Causal Effect $\theta = 0.3$			
	Mean	SE	RMSE	Type I	Mean	SE	RMSE	Coverage
IVW & Oracle	0.000	0.006	0.006	0.057	0.297	0.007	0.007	0.927
MR-Egger	0.000	0.016	0.017	0.050	0.266	0.020	0.039	0.619
Median	0.000	0.008	0.007	0.027	0.294	0.010	0.010	0.952
Mode	0.000	0.021	0.015	0.003	0.289	0.025	0.022	0.987
Lasso	0.000	0.005	0.006	0.064	0.297	0.006	0.007	0.915
MR-Presso	0.000	0.005	0.006	0.061	0.297	0.007	0.007	0.925
MR-Raps	0.000	0.006	0.006	0.051	0.300	0.007	0.007	0.962
ConMix	0.000	—	0.010	0.212	0.325	—	0.027	0.062
JAM-MR	0.000	0.006	0.006	0.060	0.295	0.007	0.008	0.904

- $N_1 = N_2 = 50000$ individuals in each GWAS and $P = 50$ genetic variants that explain 10% of the genetic variation in the risk factor. This could correspond to a Mendelian randomization analysis for a more specific risk factor, for which only a small number of genetic instruments with strong associations have been identified.
- $N_1 = N_2 = 300000$ individuals in each GWAS and $P = 50$ genetic variants that explain 3% of the genetic variation in the risk factor. This case might occur when studying a complex trait with relatively low genetic heritability.

The results of this simulation experiment are reported in Table 8. As in Table 2, the performance of JAM-MR was suboptimal in that scenario. On the other hand, increasing either the GWAS sample sizes or the proportion of genetic variation in the risk factor had a beneficial effect on the method's performance, as JAM-MR was able to identify the true causal effect with decent accuracy and its Type I error rate inflation was rather small. This suggests that JAM-MR is only unreliable in applications where both the sample size and the proportion of genetic variation in the risk factor explained by the genetic variants are small. In practice it is often possible to incorporate genetic datasets from multiple sources into a Mendelian randomization analysis, by combining consortia meta-GWAS studies with available genetic biobanks, in order to increase sample sizes. We recommend that researchers do so when using the JAM-MR algorithm.

Mild directional pleiotropy

We then generated a simulation with directional pleiotropy, but with some pleiotropic variants having a risk-increasing effect and some having a risk-decreasing effect. We did so by altering the signs of the pleiotropic effects δ_j. We used the same distribution for the effects δ_j as in the "directional pleiotropy" simulations in the main part of the paper, but randomly changed the signs of one third of these effects to the risk-decreasing direction. Given that there were 120 pleiotropic variants in our simulation, this would result in an average of 80 variants having a risk-increasing and 40 variants having a risk-decreasing pleiotropic effect on the outcome. In the simulation with violations of the InSIDE assumption, the pleiotropic effects were generated according to $\alpha_j \sim n(0.2, 0.2)$ and were risk-increasing or risk-decreasing with equal probability. Otherwise, the default parameter settings were used for this simulation.

This scenario may be called "mild directional pleiotropy" and resembles the analysis for diastolic blood pressure in the real-data application of our paper: there is some evidence of directional pleiotropy, but pleiotropic genetic variants with both risk-increasing and risk-decreasing effects are present in the dataset. Simulation results are reported in Table 9.

Similar to our previous simulations, we observed that a number of Mendelian randomization approaches were subject to pleiotropic biases. As expected, the bias was smaller than for the directional pleiotropy simulations where all pleiotropic effects were in the same direction. In fact, the differences between causal effect estimates from different methods are similar to those observed in the real-data application.

The performance of JAM-MR was similar to the simulations in the main part of the manuscript when the InSIDE assumption was satisfied, but the algorithm exhibited somewhat reduced coverage when InSIDE was violated. Note that JAM-MR does not explicitly depend on the InSIDE assumption, and its reduced coverage is probably due to the algorithm having difficulty in filtering out pleiotropic variants with relatively small pleiotropic effects (for example, variants for which the direct effects on the

TABLE 6 Simulation results with the default settings and 20% of the genetic instruments being pleiotropic.

Method	Causal Effect $\theta = 0$				Causal Effect $\theta = 0.3$			
	Mean	SE	RMSE	Type I	Mean	SE	RMSE	Coverage
**Balanced Pleiotropy**								
IVW	0.001	0.018	0.018	0.058	0.295	0.018	0.019	0.928
MR-Egger	0.005	0.054	0.055	0.062	0.262	0.055	0.069	0.888
Median	0.000	0.009	0.009	0.036	0.293	0.011	0.013	0.914
Mode	0.000	0.025	0.013	0.006	0.288	0.022	0.021	0.954
Lasso	0.000	0.006	0.007	0.080	0.295	0.007	0.010	0.842
MR-Presso	0.000	0.007	0.007	0.062	0.295	0.008	0.011	0.860
MR-Raps	0.000	0.005	0.007	0.118	0.300	0.007	0.009	0.852
ConMix	0.000	—	0.008	0.242	0.299	—	0.010	0.770
JAM-MR	0.000	0.007	0.007	0.064	0.293	0.009	0.012	0.846
Oracle	0.000	0.006	0.006	0.054	0.296	0.008	0.009	0.884
**Directional Pleiotropy, InSIDE Satisfied**								
IVW	0.140	0.017	0.140	1.000	0.437	0.017	0.137	0.000
MR-Egger	0.003	0.049	0.052	0.060	0.263	0.050	0.063	0.886
Median	0.030	0.010	0.031	0.894	0.330	0.012	0.032	0.270
Mode	0.000	0.016	0.012	0.006	0.289	0.020	0.018	0.952
Lasso	0.010	0.006	0.012	0.390	0.314	0.007	0.017	0.538
MR-Presso	0.028	0.008	0.029	0.942	0.341	0.010	0.042	0.028
MR-Raps	0.004	0.005	0.009	0.160	0.338	0.010	0.052	0.358
ConMix	0.001	—	0.008	0.198	0.302	—	0.010	0.778
JAM-MR	0.004	0.007	0.008	0.090	0.300	0.009	0.010	0.896
Oracle	0.000	0.006	0.006	0.030	0.297	0.008	0.009	0.918
**Directional Pleiotropy, InSIDE Violated**								
IVW	0.271	0.021	0.271	1.000	0.567	0.021	0.268	0.000
MR-Egger	0.577	0.057	0.580	1.000	0.856	0.058	0.559	0.000
Median	0.065	0.011	0.066	1.000	0.375	0.013	0.076	0.002
Mode	0.000	0.016	0.012	0.012	0.288	0.020	0.020	0.956
Lasso	0.010	0.006	0.012	0.356	0.313	0.008	0.017	0.580
MR-Presso	0.062	0.009	0.063	1.000	0.371	0.012	0.073	0.002
MR-Raps	0.012	0.006	0.046	0.188	0.359	0.010	0.103	0.562
ConMix	0.000	—	0.008	0.180	0.300	—	0.010	0.818
JAM-MR	0.002	0.007	0.007	0.054	0.295	0.008	0.010	0.894
Oracle	0.000	0.006	0.006	0.030	0.295	0.008	0.009	0.892

outcome and the effects on the confounder are simulated to have opposite signs). Likewise, the mode-based method performed very well in the simulations where the InSIDE assumption was satisfied, but had significantly inflated standard errors when InSIDE was violated. This performance was mostly due to a fairly small number of replications of the simulation where weak instruments were present - the median standard error across the 500 replications was 0.033 for $\theta = 0$ and 0.036 for $\theta = 0.3$. It was only observed in this simulation scenario because we generated risk-decreasing effects of genetic variants on the confounder, which have the potential to offset the variants' effects on the risk factor and appear as weak instruments.

TABLE 7 Simulation results with the default settings and 50% of the genetic instruments being pleiotropic.

Method	Causal Effect $\theta = 0$				Causal Effect $\theta = 0.3$			
	Mean	SE	RMSE	Type I	Mean	SE	RMSE	Coverage
Balanced Pleiotropy								
IVW	0.000	0.028	0.027	0.058	0.297	0.028	0.029	0.938
MR-Egger	0.008	0.082	0.085	0.058	0.261	0.083	0.090	0.930
Median	0.001	0.012	0.014	0.100	0.294	0.014	0.019	0.860
Mode	0.001	0.014	0.011	0.014	0.286	0.020	0.020	0.932
Lasso	0.001	0.008	0.010	0.122	0.296	0.009	0.013	0.832
MR-Presso	0.001	0.010	0.013	0.124	0.296	0.013	0.020	0.798
MR-Raps	0.001	0.008	0.016	0.340	0.304	0.015	0.024	0.734
ConMix	0.001	—	0.010	0.218	0.298	—	0.013	0.810
JAM-MR	0.001	0.010	0.011	0.112	0.294	0.012	0.017	0.846
Oracle	0.001	0.008	0.008	0.044	0.297	0.010	0.010	0.932
Directional Pleiotropy, InSIDE Satisfied								
IVW	0.346	0.021	0.346	1.000	0.643	0.022	0.343	0.000
MR-Egger	-0.001	0.061	0.064	0.072	0.269	0.062	0.072	0.902
Median	0.218	0.018	0.224	1.000	0.532	0.019	0.236	0.000
Mode	0.012	0.013	0.016	0.090	0.304	0.018	0.014	0.990
Lasso	0.230	0.013	0.233	1.000	0.552	0.013	0.255	0.000
MR-Presso	0.298	0.018	0.298	1.000	0.598	0.018	0.299	0.000
MR-Raps	0.339	0.023	0.339	1.000	0.640	0.024	0.341	0.000
ConMix	0.004	—	0.011	0.200	0.315	—	0.082	0.734
JAM-MR	0.007	0.010	0.013	0.142	0.307	0.013	0.034	0.862
Oracle	0.000	0.008	0.008	0.042	0.297	0.010	0.010	0.940
Directional Pleiotropy, InSIDE Violated								
IVW	0.535	0.022	0.535	1.000	0.833	0.022	0.533	0.000
MR-Egger	0.794	0.062	0.796	1.000	1.071	0.064	0.774	0.000
Median	0.618	0.015	0.618	1.000	0.909	0.018	0.610	0.000
Mode	0.017	0.017	0.094	0.028	0.302	0.027	0.081	0.966
Lasso	0.581	0.010	0.582	1.000	0.870	0.011	0.571	0.000
MR-Presso	0.556	0.018	0.556	1.000	0.846	0.019	0.546	0.000
MR-Raps	0.542	0.024	0.542	1.000	0.842	0.025	0.542	0.000
ConMix	0.001	—	0.010	0.162	1.081	—	0.835	0.138
JAM-MR	0.686	0.018	0.694	0.996	0.961	0.020	0.669	0.000
Oracle	0.001	0.008	0.008	0.038	0.296	0.010	0.011	0.922

Simulations with non-normal data

In our last set of simulations, we generated data under non-normal simulation models. Recall that our original simulation design was

$$U_i = \sum_{j=1}^{P} \alpha_j G_{ij} + \epsilon_{Ui} \tag{1}$$

$$X_i = \sum_{j=1}^{P} \beta_{Xj} G_{ij} + c_X U_i + \epsilon_{Xi} \tag{2}$$

$$Y_i = \theta X_i + \sum_{j=1}^{P} \delta_j G_{ij} + c_Y U_i + \epsilon_{Yi} \tag{3}$$

TABLE 8 Simulation results for scenario 2 (directional pleiotropy, InSIDE satisfied) with $P = 50$ SNPs and various values for the sample size and genetic variation in the risk factor.

Method	Causal Effect $\theta = 0$				Causal Effect $\theta = 0.3$			
	Mean	SE	RMSE	Type I	Mean	SE	RMSE	Coverage
$N_1 = N_2 = 50000$, $P = 50$, $GV = 3\%$								
IVW	0.205	0.057	0.208	1.000	0.497	0.061	0.202	0.030
MR-Egger	0.004	0.161	0.169	0.066	0.220	0.168	0.194	0.916
Median	0.085	0.046	0.099	0.430	0.387	0.055	0.104	0.674
Mode	0.011	0.066	0.044	0.016	0.293	0.075	0.058	0.968
Lasso	0.074	0.029	0.086	0.654	0.389	0.034	0.103	0.344
MR-Presso	0.116	0.041	0.124	0.804	0.434	0.049	0.143	0.210
MR-Raps	0.177	0.059	0.181	0.958	0.485	0.063	0.190	0.082
ConMix	0.010	—	0.045	0.102	0.351	—	0.131	0.774
JAM-MR	0.064	0.069	0.120	0.270	0.368	0.062	0.110	0.684
Oracle	-0.001	0.031	0.030	0.044	0.291	0.038	0.038	0.954
$N_1 = N_2 = 50000$, $P = 50$, $GV = 10\%$								
IVW	0.212	0.054	0.215	1.000	0.506	0.055	0.209	0.000
MR-Egger	0.010	0.164	0.172	0.058	0.267	0.164	0.180	0.916
Median	0.048	0.026	0.055	0.440	0.352	0.032	0.063	0.662
Mode	0.003	0.026	0.022	0.022	0.293	0.031	0.026	0.974
Lasso	0.023	0.016	0.031	0.320	0.328	0.019	0.040	0.670
MR-Presso	0.069	0.025	0.076	0.764	0.376	0.030	0.085	0.306
MR-Raps	0.173	0.053	0.176	0.990	0.472	0.055	0.176	0.016
ConMix	0.002	—	0.020	0.108	0.301	—	0.024	0.896
JAM-MR	0.005	0.017	0.021	0.106	0.303	0.021	0.033	0.882
Oracle	0.001	0.017	0.017	0.038	0.297	0.020	0.020	0.944
$N_1 = N_2 = 300000$, $P = 50$, $GV = 3\%$								
IVW	0.208	0.053	0.210	1.000	0.509	0.054	0.212	0.000
MR-Egger	-0.009	0.163	0.181	0.080	0.286	0.167	0.189	0.912
Median	0.037	0.020	0.043	0.414	0.342	0.025	0.052	0.638
Mode	0.000	0.018	0.016	0.032	0.294	0.022	0.021	0.974
Lasso	0.013	0.013	0.021	0.208	0.315	0.015	0.026	0.738
MR-Presso	0.066	0.022	0.073	0.818	0.365	0.025	0.074	0.294
MR-Raps	0.166	0.052	0.170	0.982	0.469	0.053	0.173	0.014
ConMix	0.001	—	0.014	0.100	0.299	—	0.019	0.876
JAM-MR	0.001	0.013	0.014	0.088	0.298	0.015	0.019	0.886
Oracle	0.000	0.013	0.013	0.040	0.297	0.016	0.016	0.934

where $\epsilon_{Ui} \sim N(0, \sigma^2_{\epsilon_U})$, $\epsilon_{Xi} \sim N(0, \sigma^2_{\epsilon_X})$, $\epsilon_{Yi} \sim N(0, \sigma^2_{\epsilon_Y})$ independently of each other. To assess the robustness of the various methods to violations of normality assumptions, we performed three additional sets of simulations, in each case modifying the distribution of one of the three residuals ϵ_U, ϵ_X, ϵ_Y. That way we could assess the impact of violations of the normality assumptions about the confounder, risk factor and outcome separately. Two non-normal distributions were used for each residual:

- A t distribution with 5 degrees of freedom, rescaled to have variance σ^2_*, or
- A Uniform distribution in the interval $(-2\sigma^2_*, 2\sigma^2_*$.

where σ^2_* is the variance of the corresponding residual (that is, $\sigma^2_* = \sigma^2_{\epsilon_U}$ for the confounder residual ϵ_U, $\sigma^2_* = \sigma^2_{\epsilon_X}$ for the risk factor residual ϵ_X, and $\sigma^2_* = \sigma^2_{\epsilon_Y}$ for the outcome residual ϵ_Y.

TABLE 9 Simulation results with sample sizes of $N_1 = N_2 = 50000$ and $P = 50$ genetic variants that explain 3% genetic variation in the risk factor.

Method	Causal Effect $\theta = 0$				Causal Effect $\theta = 0.3$			
	Mean	SE	RMSE	Type I	Mean	SE	RMSE	Coverage
InSIDE Satisfied								
IVW	0.070	0.021	0.073	0.906	0.364	0.022	0.067	0.138
MR.Egger	-0.003	0.063	0.066	0.046	0.259	0.065	0.078	0.884
Median	0.016	0.010	0.019	0.354	0.313	0.012	0.018	0.804
Mode	0.000	0.017	0.012	0.020	0.287	0.029	0.020	0.958
Lasso	0.006	0.007	0.009	0.158	0.306	0.008	0.011	0.824
MR.Presso	0.013	0.008	0.016	0.390	0.319	0.010	0.023	0.518
MR.Raps	0.059	0.019	0.063	0.864	0.360	0.020	0.063	0.128
ConMix	0.000	—	0.008	0.200	0.299	—	0.011	0.804
JAM.MR	0.002	0.007	0.009	0.082	0.297	0.010	0.012	0.882
Oracle	0.000	0.007	0.000	0.044	0.296	0.008	0.004	0.922
InSIDE Violated								
IVW	0.094	0.022	0.096	0.990	0.391	0.023	0.094	0.028
MR.Egger	0.181	0.061	0.195	0.784	0.454	0.062	0.172	0.362
Median	0.020	0.010	0.022	0.496	0.318	0.012	0.022	0.652
Mode	0.003	0.801	0.382	0.004	0.293	0.794	0.123	0.990
Lasso	0.006	0.006	0.009	0.198	0.306	0.008	0.012	0.804
MR.Presso	0.015	0.008	0.017	0.458	0.318	0.010	0.022	0.570
MR.Raps	0.012	0.007	0.028	0.328	0.331	0.013	0.044	0.456
ConMix	0.000	—	0.008	0.218	0.297	—	0.011	0.736
JAM.MR	-0.002	0.008	0.009	0.106	0.291	0.010	0.015	0.772
Oracle	0.001	0.007	0.000	0.040	0.296	0.008	0.004	0.918

Simulation results are presented in Tables 10-11 for the confounder residual, Tables 12-13 for the risk factor residual, and Tables 14-15 for the outcome residual.

A comparison of the simulation results in Tables 10-15 with those in Table 2 in the main part of our paper suggests that even though many of the Mendelian randomization methods considered make normality assumptions about the risk factor and/or the outcome, in practice violations of these assumptions are less concerning than bias due to pleiotropy. In fact, most methods performed almost as well in these non-normal simulations as in our original simulation. Based on this simulation experiment, it is not clear which methods are more sensitive to violations of normality assumptions.

MANHATTAN PLOTS FOR THE REAL-DATA APPLICATION

Here we provide Manhattan plots obtained by running JAM-MR for the application in Section 4 of the main paper. Two Manhattan plots are given: one for systolic and one for diastolic blood pressure. In separate files, we provide the corresponding tables of results. These contain SNP names and chromosome positions, univariate summary statistics of association between SNPs and blood pressure traits, as well as coronary heart disease, and posterior inclusion probabilities assigned by JAM-MR to each SNP.

TABLE 10 Simulation results for simulations where $\epsilon_U \sim t(5)$.

Method	Causal Effect $\theta = 0$				Causal Effect $\theta = 0.3$			
	Mean	SE	RMSE	Type I	Mean	SE	RMSE	Coverage
Balanced Pleiotropy								
IVW	-0.002	0.022	0.021	0.034	0.297	0.022	0.023	0.946
MR.Egger	-0.007	0.064	0.065	0.050	0.259	0.065	0.078	0.884
Median	-0.001	0.011	0.012	0.048	0.293	0.014	0.017	0.906
Mode	-0.001	0.020	0.014	0.010	0.284	0.031	0.024	0.968
Lasso	0.000	0.007	0.009	0.076	0.295	0.009	0.013	0.838
MR.Presso	0.000	0.009	0.010	0.086	0.296	0.012	0.016	0.868
MR.Raps	-0.002	0.020	0.018	0.040	0.302	0.021	0.021	0.950
ConMix	0.000	—	0.009	0.208	0.298	—	0.013	0.828
JAM.MR	0.000	0.009	0.010	0.086	0.291	0.012	0.016	0.850
Oracle	0.000	0.008	0.000	0.042	0.295	0.009	0.005	0.922
Directional Pleiotropy, InSIDE Satisfied								
IVW	0.207	0.019	0.208	1.000	0.501	0.020	0.201	0.000
MR.Egger	0.004	0.055	0.055	0.046	0.255	0.057	0.073	0.868
Median	0.060	0.012	0.062	1.000	0.366	0.015	0.068	0.006
Mode	0.002	0.024	0.030	0.010	0.288	0.026	0.020	0.974
Lasso	0.038	0.008	0.040	0.978	0.353	0.009	0.055	0.008
MR.Presso	0.091	0.012	0.092	1.000	0.415	0.015	0.116	0.000
MR.Raps	0.173	0.020	0.173	1.000	0.475	0.020	0.175	0.000
ConMix	0.002	—	0.010	0.230	0.305	—	0.014	0.784
JAM.MR	0.007	0.009	0.013	0.156	0.303	0.012	0.015	0.894
Oracle	0.000	0.008	0.000	0.062	0.295	0.009	0.005	0.912
Directional Pleiotropy, InSIDE Violated								
IVW	0.370	0.022	0.371	1.000	0.666	0.023	0.366	0.000
MR-Egger	0.688	0.061	0.691	1.000	0.956	0.062	0.659	0.000
Median	0.187	0.020	0.195	1.000	0.522	0.023	0.228	0.000
Mode	0.000	0.016	0.012	0.010	0.285	0.024	0.022	0.934
Lasso	0.059	0.009	0.061	1.000	0.389	0.011	0.091	0.000
MR-Presso	0.211	0.017	0.212	1.000	0.534	0.018	0.235	0.000
MR-Raps	0.338	0.024	0.339	1.000	0.640	0.025	0.340	0.000
ConMix	0.000	—	0.009	0.176	0.300	—	0.012	0.850
JAM-MR	0.003	0.009	0.009	0.056	0.294	0.012	0.014	0.902
Oracle	0.000	0.008	0.000	0.034	0.294	0.010	0.006	0.922

TABLE 11 Simulation results for simulations where $\epsilon_U \sim U(-2\sigma_U^2, 2\sigma_U^2)$.

Method	Causal Effect $\theta = 0$				Causal Effect $\theta = 0.3$			
	Mean	SE	RMSE	Type I	Mean	SE	RMSE	Coverage
Balanced Pleiotropy								
IVW	0.000	0.022	0.022	0.054	0.296	0.022	0.022	0.944
MR.Egger	0.001	0.064	0.072	0.074	0.262	0.066	0.075	0.926
Median	0.000	0.011	0.011	0.060	0.293	0.013	0.015	0.908
Mode	-0.001	0.017	0.013	0.008	0.287	0.022	0.022	0.962
Lasso	0.000	0.007	0.008	0.092	0.295	0.008	0.012	0.842
MR.Presso	0.000	0.008	0.009	0.080	0.296	0.011	0.014	0.848
MR.Raps	0.001	0.020	0.018	0.032	0.301	0.021	0.019	0.974
ConMix	0.000	—	0.009	0.208	0.298	—	0.012	0.806
JAM.MR	0.000	0.008	0.009	0.086	0.292	0.011	0.014	0.844
Oracle	0.000	0.007	0.000	0.046	0.295	0.009	0.005	0.912
Directional Pleiotropy, InSIDE Satisfied								
IVW	0.207	0.019	0.207	1.000	0.502	0.020	0.203	0.000
MR.Egger	0.004	0.056	0.056	0.050	0.261	0.057	0.068	0.916
Median	0.056	0.012	0.057	1.000	0.363	0.014	0.064	0.000
Mode	0.001	0.015	0.011	0.014	0.288	0.020	0.019	0.958
Lasso	0.033	0.007	0.034	0.974	0.346	0.009	0.048	0.000
MR.Presso	0.083	0.011	0.084	1.000	0.406	0.014	0.107	0.000
MR.Raps	0.171	0.019	0.172	1.000	0.474	0.020	0.175	0.000
ConMix	0.001	—	0.009	0.202	0.304	—	0.013	0.794
JAM.MR	0.005	0.008	0.011	0.110	0.302	0.011	0.014	0.882
Oracle	0.000	0.007	0.000	0.042	0.296	0.009	0.004	0.924
Directional Pleiotropy, InSIDE Violated								
IVW	0.372	0.022	0.372	1.000	0.666	0.023	0.367	0.000
MR-Egger	0.701	0.060	0.704	1.000	0.961	0.062	0.664	0.000
Median	0.182	0.019	0.191	1.000	0.508	0.022	0.215	0.000
Mode	0.000	0.014	0.011	0.012	0.287	0.020	0.019	0.950
Lasso	0.053	0.008	0.055	0.998	0.378	0.010	0.080	0.000
MR-Presso	0.207	0.017	0.209	1.000	0.525	0.018	0.226	0.000
MR-Raps	0.340	0.024	0.340	1.000	0.639	0.024	0.340	0.000
ConMix	0.000	—	0.009	0.176	0.301	—	0.012	0.834
JAM-MR	0.003	0.008	0.009	0.070	0.297	0.010	0.012	0.896
Oracle	0.000	0.007	0.000	0.032	0.296	0.009	0.004	0.922

TABLE 12 Simulation results for simulations where $\epsilon_X \sim t(5)$.

Method	Causal Effect $\theta = 0$				Causal Effect $\theta = 0.3$			
	Mean	SE	RMSE	Type I	Mean	SE	RMSE	Coverage
Balanced Pleiotropy								
IVW	0.001	0.022	0.022	0.062	0.295	0.022	0.022	0.958
MR.Egger	0.004	0.064	0.065	0.060	0.257	0.064	0.078	0.888
Median	0.000	0.010	0.010	0.052	0.291	0.012	0.015	0.900
Mode	0.001	0.020	0.013	0.006	0.284	0.020	0.022	0.926
Lasso	0.000	0.007	0.008	0.092	0.295	0.008	0.011	0.834
MR.Presso	0.000	0.007	0.008	0.080	0.295	0.010	0.012	0.874
MR.Raps	0.000	0.020	0.019	0.044	0.300	0.020	0.019	0.970
ConMix	0.001	—	0.008	0.204	0.296	—	0.011	0.774
JAM.MR	0.000	0.008	0.009	0.088	0.291	0.010	0.014	0.804
Oracle	0.000	0.007	0.000	0.046	0.295	0.008	0.005	0.892
Directional Pleiotropy, InSIDE Satisfied								
IVW	0.206	0.019	0.206	1.000	0.501	0.019	0.202	0.000
MR.Egger	-0.001	0.055	0.058	0.066	0.257	0.056	0.070	0.890
Median	0.052	0.011	0.053	1.000	0.357	0.014	0.059	0.008
Mode	0.000	0.017	0.011	0.012	0.287	0.031	0.023	0.942
Lasso	0.028	0.007	0.029	0.954	0.340	0.008	0.042	0.018
MR.Presso	0.077	0.011	0.078	1.000	0.399	0.013	0.100	0.000
MR.Raps	0.170	0.019	0.170	1.000	0.474	0.020	0.174	0.000
ConMix	0.001	—	0.009	0.236	0.301	—	0.010	0.802
JAM.MR	0.005	0.008	0.010	0.150	0.299	0.010	0.012	0.898
Oracle	0.000	0.007	0.000	0.048	0.295	0.008	0.005	0.912
Directional Pleiotropy, InSIDE Violated								
IVW	0.371	0.022	0.371	1.000	0.665	0.023	0.366	0.000
MR-Egger	0.687	0.060	0.689	1.000	0.952	0.062	0.655	0.000
Median	0.164	0.018	0.172	1.000	0.495	0.022	0.202	0.000
Mode	0.001	0.019	0.021	0.008	0.285	0.021	0.021	0.910
Lasso	0.045	0.008	0.046	1.000	0.368	0.009	0.070	0.000
MR-Presso	0.202	0.017	0.204	1.000	0.519	0.018	0.220	0.000
MR-Raps	0.339	0.024	0.339	1.000	0.639	0.024	0.339	0.000
ConMix	0.000	—	0.008	0.200	0.299	—	0.010	0.838
JAM-MR	0.002	0.007	0.008	0.058	0.295	0.010	0.011	0.900
Oracle	0.000	0.007	0.000	0.046	0.295	0.008	0.005	0.908

TABLE 13 Simulation results for simulations where $\epsilon_X \sim U(-2\sigma_X^2, 2\sigma_X^2)$.

Method	Causal Effect $\theta = 0$				Causal Effect $\theta = 0.3$			
	Mean	SE	RMSE	Type I	Mean	SE	RMSE	Coverage
Balanced Pleiotropy								
IVW	0.001	0.022	0.022	0.054	0.297	0.022	0.023	0.926
MR.Egger	0.001	0.064	0.065	0.056	0.260	0.065	0.078	0.888
Median	-0.001	0.010	0.011	0.076	0.293	0.012	0.015	0.892
Mode	-0.001	0.015	0.013	0.018	0.286	0.020	0.021	0.958
Lasso	0.000	0.007	0.007	0.076	0.295	0.008	0.011	0.836
MR.Presso	0.000	0.007	0.009	0.082	0.295	0.010	0.013	0.852
MR.Raps	0.001	0.020	0.019	0.052	0.301	0.020	0.020	0.940
ConMix	0.000	—	0.008	0.232	0.297	—	0.011	0.760
JAM.MR	0.000	0.008	0.009	0.090	0.291	0.010	0.014	0.818
Oracle	0.000	0.007	0.000	0.046	0.295	0.008	0.005	0.914
Directional Pleiotropy, InSIDE Satisfied								
IVW	0.208	0.019	0.208	1.000	0.503	0.020	0.204	0.000
MR.Egger	0.003	0.055	0.061	0.072	0.262	0.057	0.070	0.876
Median	0.053	0.011	0.054	0.998	0.357	0.013	0.059	0.010
Mode	0.001	0.014	0.011	0.016	0.290	0.028	0.043	0.964
Lasso	0.028	0.007	0.030	0.948	0.339	0.008	0.041	0.018
MR.Presso	0.077	0.011	0.078	1.000	0.398	0.013	0.099	0.000
MR.Raps	0.171	0.019	0.171	1.000	0.474	0.020	0.175	0.000
ConMix	0.002	—	0.009	0.208	0.302	—	0.011	0.816
JAM.MR	0.005	0.008	0.010	0.118	0.300	0.010	0.011	0.918
Oracle	0.000	0.007	0.000	0.038	0.296	0.008	0.004	0.922
Directional Pleiotropy, InSIDE Violated								
IVW	0.371	0.022	0.371	1.000	0.666	0.023	0.367	0.000
MR-Egger	0.695	0.060	0.698	1.000	0.960	0.062	0.663	0.000
Median	0.163	0.018	0.171	1.000	0.493	0.021	0.200	0.000
Mode	0.000	0.013	0.010	0.012	0.285	0.020	0.020	0.896
Lasso	0.043	0.008	0.045	0.992	0.367	0.009	0.069	0.000
MR-Presso	0.202	0.017	0.204	1.000	0.517	0.018	0.218	0.000
MR-Raps	0.338	0.024	0.338	1.000	0.639	0.024	0.339	0.000
ConMix	-0.001	—	0.008	0.180	0.299	—	0.010	0.816
JAM-MR	0.001	0.007	0.008	0.068	0.295	0.009	0.012	0.876
Oracle	-0.001	0.007	0.001	0.048	0.295	0.008	0.005	0.910

TABLE 14 Simulation results for simulations where $\epsilon_Y \sim t(5)$.

Method	Causal Effect $\theta = 0$				Causal Effect $\theta = 0.3$			
	Mean	SE	RMSE	Type I	Mean	SE	RMSE	Coverage
**Balanced Pleiotropy**								
IVW	-0.001	0.022	0.023	0.062	0.297	0.022	0.023	0.938
MR.Egger	-0.002	0.065	0.068	0.066	0.266	0.066	0.076	0.918
Median	-0.001	0.011	0.013	0.088	0.295	0.013	0.015	0.916
Mode	-0.001	0.018	0.015	0.018	0.289	0.022	0.020	0.976
Lasso	-0.001	0.008	0.009	0.104	0.297	0.009	0.011	0.872
MR.Presso	-0.001	0.009	0.010	0.102	0.297	0.011	0.014	0.868
MR.Raps	-0.001	0.020	0.020	0.052	0.301	0.021	0.020	0.948
ConMix	-0.001	0.000	0.010	0.200	0.300	0.000	0.012	0.822
JAM.MR	-0.001	0.009	0.010	0.072	0.294	0.011	0.015	0.840
Oracle	-0.000	0.008	0.000	0.046	0.297	0.009	0.003	0.922
**Directional Pleiotropy, InSIDE Satisfied**								
IVW	0.207	0.019	0.208	1.000	0.503	0.020	0.204	0.000
MR.Egger	-0.006	0.056	0.056	0.054	0.266	0.058	0.068	0.902
Median	0.061	0.012	0.062	0.998	0.364	0.014	0.066	0.000
Mode	0.002	0.017	0.014	0.018	0.290	0.021	0.018	0.984
Lasso	0.039	0.008	0.040	0.988	0.347	0.009	0.049	0.006
MR.Presso	0.060	0.012	0.091	1.000	0.407	0.014	0.108	0.000
MR.Raps	0.172	0.019	0.172	1.000	0.474	0.020	0.174	0.000
ConMix	0.004	0.000	0.010	0.204	0.305	0.000	0.013	0.812
JAM.MR	0.008	0.009	0.013	0.172	0.303	0.011	0.014	0.892
Oracle	0.001	0.008	0.001	0.070	0.296	0.009	0.004	0.930
**Directional Pleiotropy, InSIDE Violated**								
IVW	0.372	0.022	0.372	1.000	0.668	0.023	0.368	0.000
MR-Egger	0.706	0.061	0.708	1.000	0.977	0.062	0.680	0.000
Median	0.190	0.020	0.197	1.000	0.516	0.022	0.223	0.000
Mode	0.000	0.015	0.012	0.012	0.288	0.019	0.019	0.936
Lasso	0.060	0.009	0.062	1.000	0.380	0.010	0.081	0.000
MR-Presso	0.211	0.017	0.212	1.000	0.527	0.018	0.229	0.000
MR-Raps	0.340	0.024	0.340	1.000	0.640	0.024	0.340	0.000
ConMix	0.000	—	0.010	0.188	0.301	—	0.012	0.850
JAM-MR	0.003	0.009	0.010	0.098	0.296	0.011	0.012	0.912
Oracle	0.000	0.008	0.000	0.046	0.295	0.009	0.005	0.918

TABLE 15 Simulation results for simulations where $\epsilon_Y \sim U(-2\sigma_Y^2, 2\sigma_Y^2)$.

Method	Causal Effect $\theta = 0$				Causal Effect $\theta = 0.3$			
	Mean	SE	RMSE	Type I	Mean	SE	RMSE	Coverage
Balanced Pleiotropy								
IVW	-0.002	0.022	0.023	0.082	0.296	0.022	0.025	0.924
MR.Egger	-0.004	0.065	0.067	0.056	0.261	0.066	0.079	0.894
Median	0.000	0.011	0.011	0.062	0.293	0.013	0.015	0.882
Mode	0.000	0.017	0.013	0.012	0.288	0.028	0.032	0.952
Lasso	0.000	0.007	0.008	0.100	0.295	0.008	0.011	0.834
MR.Presso	0.000	0.008	0.009	0.088	0.296	0.010	0.014	0.824
MR.Raps	-0.001	0.020	0.020	0.050	0.300	0.020	0.022	0.942
ConMix	0.000	0.000	0.009	0.200	0.298	0.000	0.011	0.774
JAM.MR	0.001	0.008	0.009	0.084	0.293	0.010	0.015	0.814
Oracle	0.000	0.007	0.000	0.038	0.296	0.008	0.004	0.920
Directional Pleiotropy, InSIDE Satisfied								
IVW	0.207	0.019	0.207	1.000	0.503	0.020	0.204	0.000
MR.Egger	-0.001	0.056	0.056	0.048	0.263	0.057	0.069	0.906
Median	0.057	0.012	0.058	0.998	0.361	0.014	0.063	0.002
Mode	0.001	0.016	0.011	0.006	0.289	0.020	0.018	0.970
Lasso	0.033	0.007	0.035	0.982	0.345	0.009	0.046	0.008
MR.Presso	0.083	0.011	0.085	1.000	0.402	0.014	0.103	0.000
MR.Raps	0.171	0.019	0.171	1.000	0.473	0.020	0.174	0.000
ConMix	0.002	0.000	0.009	0.182	0.304	0.000	0.012	0.784
JAM.MR	0.006	0.008	0.011	0.134	0.302	0.010	0.013	0.896
Oracle	0.000	0.007	0.000	0.046	0.296	0.009	0.004	0.956
Directional Pleiotropy, InSIDE Violated								
IVW	0.372	0.022	0.372	1.000	0.670	0.023	0.370	0.000
MR-Egger	0.708	0.061	0.711	1.000	0.984	0.062	0.687	0.000
Median	0.176	0.019	0.183	1.000	0.506	0.022	0.213	0.000
Mode	0.000	0.014	0.011	0.018	0.289	0.018	0.018	0.940
Lasso	0.052	0.008	0.053	1.000	0.374	0.010	0.076	0.000
MR-Presso	0.206	0.017	0.208	1.000	0.524	0.018	0.225	0.000
MR-Raps	0.340	0.024	0.340	1.000	0.641	0.024	0.342	0.000
ConMix	0.000	—	0.009	0.182	0.301	—	0.012	0.798
JAM-MR	0.002	0.008	0.009	0.064	0.297	0.010	0.011	0.914
Oracle	0.000	0.007	0.000	0.040	0.296	0.009	0.004	0.934

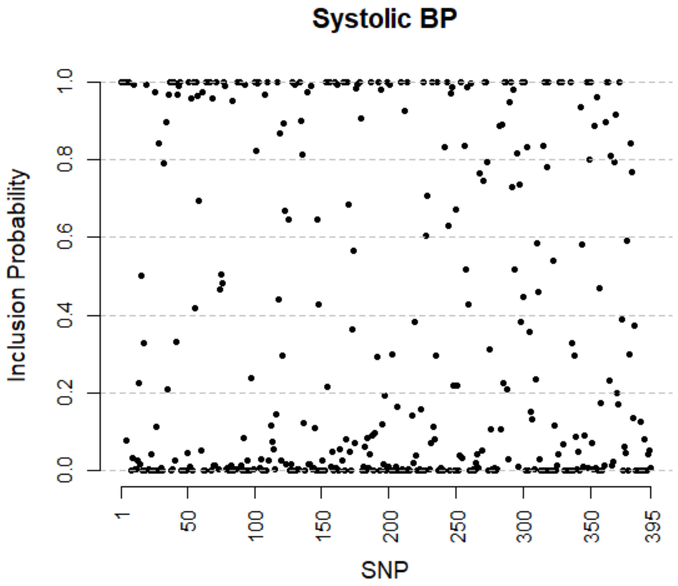

FIGURE 3 Manhattan plot of posterior inclusion probabilities for the 395 SBP-associated genetic variants.

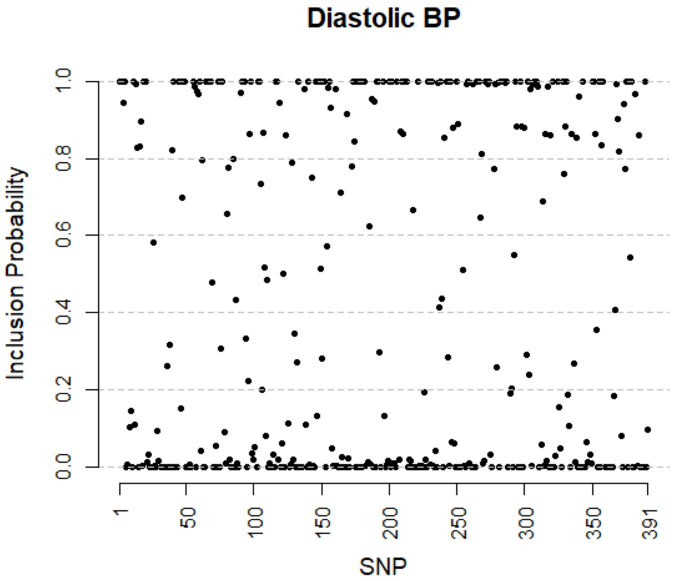

FIGURE 4 Manhattan plot of posterior inclusion probabilities for the 391 DBP-associated genetic variants.

References

1. Bowden J, Davey Smith G, Burgess S. Mendelian randomization with invalid instruments: effect estimation and bias detection through Egger regression. *International Journal of Epidemiology* 2015; 44(2): 512–525. doi: 10.1093/ije/dyv080

2. Hartwig FP, Davies NM, Hemani G, Davey Smith G. Two-sample Mendelian randomization: avoiding the downsides of a powerful, widely applicable but potentially fallible technique. *International Journal of Epidemiology* 2016; 45(6): 1717-1726. doi: 10.1093/ije/dyx028

3. Kang H, Zhang A, Cai T, Small D. Instrumental variables estimation with some invalid instruments, and its application to Mendelian randomisation. *Journal of the American Statistical Association* 2016; 111(513): 132–144. doi: 10.1080/01621459.2014.994705

4. Rees JMB, Wood AM, Dudbridge F, Burgess S. Robust methods in Mendelian randomization via penalization of heterogeneous causal estimates. *PLoS ONE* 2019; 14(9): 1-24. doi: 10.1371/journal.pone.0222362

5. Verbanck M, Chen CY, Neele B, Do R. Detection of widespread horizontal pleiotropy in causal relationships inferred from Mendelian randomization between complex traits and diseases. *Nature Genetics* 2018; 50(3): 693-698.

6. Burgess S, Foley CN, Allara E, Staley JR, Howson JMM. A robust and efficient method for Mendelian randomization with hundreds of genetic variants: unravelling mechanisms linking HDL-cholesterol and coronary heart disease. *bioRxiv* 2019. doi: 10.1101/566851

7. Zhao Q, Wang J, Hemani G, Bowden J, Small DS. Statistical inference in two-sample summary-data Mendelian randomization using robust adjusted profile score. arXiv:1801.09652v2; 2018.

8. Slob EA, Burgess S. A Comparison Of Robust Mendelian Randomization Methods Using Summary Data. *bioRxiv* 2019. doi: 10.1101/577940

9. Qi G, Chatterjee N. A Comprehensive Evaluation of Methods for Mendelian Randomization Using Realistic Simulations of Genome-wide Association Studies. *bioRxiv* 2019. doi: 10.1101/702787

10. Burgess S, Zuber V, Gkatzionis A, Foley CN. Modal-based estimation via heterogeneity-penalized weighting: model averaging for consistent and efficient estimation in Mendelian randomization when a plurality of candidate instruments are valid. *International Journal of Epidemiology* 2018; 47(4): 1242-1254. doi: 10.1093/ije/dyy080

11. Qi G, Chatterjee N. Mendelian Randomization Analysis Using Mixture Models (MRMix) for Genetic Effect-Size-Distribution Leads to Robust Estimation of Causal Effects. bioRxiv, doi:10.1101/367821; 2018.

12. Koop G, Leon-Gonzalez R, Strachan R. Bayesian model averaging in the instrumental variable regression model. *Journal of Econometrics* 2012; 171(2): 237 - 250. Bayesian Models, Methods and Applicationsdoi: http://dx.doi.org/10.1016/j.jeconom.2012.06.005

13. Burgess S, Bowden J. Integrating summarized data from multiple genetic variants in Mendelian randomization: bias and coverage properties of inverse-variance weighted methods. https://arxiv.org/abs/1512.04486v1; 2016.

□
